# BPZE1 vaccination induces IL-17^+^ and IL-22^+^ CD4^+^ T cells associated with nasal mucosal secretory IgA responses in humans: effects on virulent *Bordetella pertussis* colonisation

**DOI:** 10.1038/s41541-026-01443-7

**Published:** 2026-04-18

**Authors:** Alison R. Hill, Diane F. Gbesemete, Tyween Coutinho, Muktar Ibrahim, Jay R. Laver, Peter Goldstein, Stephanie Noviello, Keith Rubin, Saul N. Faust, Camille Locht, Robert C. Read, Adam P. Dale

**Affiliations:** 1 Controlled Human Infection Group, School of Clinical & Experimental Sciences, Faculty of Medicine, University of Southampton, Southampton, UK; 2 NIHR Southampton Clinical Research Facility and Biomedical Research Centre, University Hospital Southampton NHS Foundation Trust, Southampton, UK; 3 ILiAD Biotechnologies, Weston, FL USA; 4 Faculty of Medicine and Institute for Life Sciences, University of Southampton, Southampton, UK; 5 Univ. Lille, CNRS, Inserm, CHU Lille, Institut Pasteur de Lille, U1019-UMR9017–CIIL - Centre for Infection and Immunity of Lille, Lille, France

**Keywords:** Diseases, Immunology, Microbiology

## Abstract

Pertussis remains a major public health challenge due to the inability of current acellular vaccines to prevent *Bordetella pertussis* colonisation and transmission. The live-attenuated intranasal vaccine, BPZE1, has shown efficacy in protecting against infection following challenge with virulent *B. pertussis,* but the immune mechanisms underpinning this protective effect are poorly defined. In this study, we demonstrate that intranasal vaccination with BPZE1 induced circulating CD4^+^ T cells of Th17 and Th22 effector phenotype. These responses correlated positively with BPZE1-induced secretory IgA (SIgA) responses, suggesting coordinated induction of mucosal immunity. Among BPZE1 recipients who developed breakthrough infection following virulent *B. pertussis* challenge, higher vaccine-induced SIgA titres were significantly associated with reduced colonisation density. By contrast, BPZE1-induced serum IgA and IgG titres were not associated with reduced colonisation density. These findings identify a Th17/Th22-SIgA immune axis as a feature of BPZE1 vaccination and support the hypothesis that BPZE1-induced SIgA may contribute to reduced *B. pertussis* colonisation density in participants with virulent *B. pertussis* breakthrough infection despite BPZE1 vaccination.

## Introduction

Pertussis is a highly contagious infectious disease caused by *Bordetella pertussis*. Although infection occurs across the life course, the burden of severe disease falls disproportionately on young children^1^. Despite widespread use of acellular pertussis (aP) vaccines, periodic surges of pertussis continue to occur, likely due to rapidly waning immunity, pathogen adaptation, and the inability of aP to protect against colonisation or transmission^2–5^. In a baboon model, aP vaccination failed to prevent *B. pertussis* colonisation and transmission^6^, and epidemiological modelling suggests that asymptomatic transmission is a key driver of pertussis resurgence^5^. Asymptomatic *B. pertussis* colonisation in adults has also been demonstrated in a controlled human infection model^7^. Improved control of pertussis at the population level will require vaccines capable of inducing herd immunity through the prevention of nasopharyngeal colonisation and transmission.

Preclinical studies suggest that the live-attenuated vaccine candidate BPZE1 protects against *B. pertussis* nasal colonisation through an IL-17-dependent, SIgA-mediated mechanism^8^, and that BPZE1 induces CD4^+^ T cell responses with a mixed Th1/Th17 effector phenotype^9,10^. In humans, BPZE1 has been shown to elicit nasal mucosal SIgA, serum IgA, and serum IgG responses to *B. pertussis* antigens^11–13^. However, the effector phenotype of circulating BPZE1-induced CD4^+^ T cells was hitherto undefined.

Recently, a randomised, placebo-controlled trial demonstrated that intranasal BPZE1 vaccination can protect against virulent *B. pertussis* colonisation in a controlled human infection model^13^. Here, we assessed the frequency and effector phenotype of T cells induced by BPZE1 vaccination in the setting of this trial, testing the hypothesis that BPZE1 induces a Th1/Th17-polarised CD4^+^ T cell response, and sought to determine whether BPZE1-induced *Bp*-specific CD4^+^ T cell or humoral immunodynamics were linked to protection against colonisation following intranasal inoculation with virulent *B. pertussis*.

## Methods

### Study design, participants and sampling

Full details relating to the clinical study design for this two-site, randomised placebo-controlled Phase 2b vaccine-challenge trial, including data pertaining to participant demographics, the primary outcome and safety, are published elsewhere^13^. This study adhered to the 2013 Helsinki Declaration, was approved by the London Central Research Ethics Committee (22/FT/006) and is registered with ClinicalTrials.gov, NCT05461131 (posted 15/07/2022, now completed). Written informed consent was obtained from all participants.

In brief, eligible participants with baseline anti-pertussis toxin (PT) and anti-pertactin (PRN) serum IgG titres ≤20 IU ml^−1^ and ≤30 IU ml^−1^, respectively, were randomly assigned (1:1) to receive an intranasal dose of 10^9^ colony-forming units (CFU) of BPZE1 or placebo. BPZE1 and placebo were reconstituted in 1 ml sterile water and administered 0.4 ml per nostril by the intranasal mucosal atomisation device (MAD Nasal; Teleflex). Sixty to 120 days post-vaccination, following clinical examination and negative *B. pertussis* culture, participants were challenged with 10^5^ CFU virulent *B. pertussis* B1917, a fully genotyped representative of current isolates in Europe^14^, as droplets into each nostril administered using a P1000 micropipette (StarLabs), as described^13^.

### Culture of *B*. *pertussis* and determination of colonisation density

Assessment of virulent *B. pertussis* colonisation was performed on days 9, 11 and 14 post-challenge using quantitative culture of nasal wash samples on charcoal agar with detection limits between 4 and 123,600 CFU^7,13^. *B*. *pertussis* colonisation density was estimated by calculation of the area under the curve (AUC) based on absolute counts at post-challenge days 9, 11 and 14, as described in ref. ^13^.

### Whole blood T cell assay

The frequency and effector phenotype of CD4^+^ T cells with specificity to the *B. pertussis* antigens filamentous haemagglutinin (FHA – Native Antigen Company) and heat-inactivated PT (Native Antigen Company) were assessed in fresh whole blood collected in 10 ml sodium heparin blood collection tubes (BD Biosciences), utilising a previously validated assay^15^. Briefly, whole blood samples were diluted 1:1 with RPMI 1640 + GlutaMax medium (Gibco) supplemented with penicillin (100 U ml^−1^) and streptomycin (100 µg ml^−1^) (Gibco), and incubated at 37 °C, 5% CO_2,_ in 15 ml round bottom tubes (Falcon) (800 µl per tube) in the absence (negative control) or presence of 1 µg ml^−1^ *Staphylococcus* enterotoxin B (SEB) (Sigma-Aldrich), 5 µg ml^−1^ PT, or 5 µg ml^−1^ FHA. Co-stimulatory antibody cocktail (1 µg ml^−1^ anti-CD28 and 1 µg ml^−1^ anti-CD49d) (BD Biosciences) was added to all stimulation conditions except for SEB. Following a 19-h incubation, cellular protein transport was inhibited by the addition of 10 µg ml^−1^ Brefeldin A (Sigma-Aldrich) and 1:1000 dilution of GolgiStop (BD Biosciences) and the cells were incubated for a further 5 h. Subsequently, erythrocytes were lysed by 10-min incubation with 10 ml Pharmlyse (BD Biosciences) at room temperature. Cells were then pelleted (500 × *g* for 10 min), washed with 10 ml phosphate-buffered saline (PBS) (Severn Biotech), pelleted again by centrifugation (500 × *g* for 10 min), and fixed by incubation with 1 ml Cytofix/Cytoperm solution (BD Biosciences) for 20 min at room temperature. After further centrifugation (500 × *g* for 5 min), cells were cryopreserved in vapour phase liquid nitrogen in 500 µl Recovery Cell Culture Freezing Medium (Gibco).

Cryopreserved cells were subsequently thawed in batches for each participant and permeabilised by 10-min incubation with 2 ml Perm/Wash solution (BD Biosciences) at room temperature in 3 ml FACS tubes (Falcon). Samples were pelleted by centrifugation at 500 × *g* for 5 min before the addition of 100 µl antibody mix for intracellular antibody staining. Intracellular antibody staining was performed in the presence of Perm/Wash solution for 30 min at room temperature with the following antibody panel: CD4-BB515 (BD Biosciences, clone SK3, 2.5 µl), IL-17A-PE (Biolegend, clone BL168, 2.5 µl), IL-17F-PE (BD Biosciences, clone 033-782, 5 µl), IL-22-PeCy7 (eBiosciences, clone 22URTI, 5 µl), CD8-Percp-Cy5.5 (BD Biosciences, clone SK1, 2.5 µl), IL-4-APC (Biolegend, clone MP-4-25D2, 5 µl), IL-5-APC (Biolegend, clone TRKF5, 5 µl), IL-13-APC (Biolegend, clone JES105A2, 5 µl), CD3-APC-H7 (BD Biosciences, clone SK7, 2.5 µl), IFN-γ-BV421 (BD Biosciences, clone B27, 2.5 µl) (Supplementary Table 1). After antibody staining, cells were washed with 2 ml Perm/Wash solution, pelleted by centrifugation (500 × *g* for 5 min) and then washed with 2 ml FACS wash solution (1% Bovine Serum Albumin [Europa Bioproducts], 0.05% sodium azide [Merck] in PBS), before pelleting (500 × *g* for 5 min) and resuspending in a final volume of 200 µl in FACS wash solution.

The complete contents of each tube were acquired on the FACS ARIA IIu instrument (BD Biosciences). Compensation controls were prepared using compensation beads (BD Biosciences) individually stained with each monoclonal antibody-fluorophore conjugate, with compensation calculated using BD FACSDiva 8 software. Flow cytometry data were analysed using FlowJo software (v10.10, BD Biosciences), using a sequential gating strategy as outlined in Supplementary Fig. 1. Briefly, lymphocytes were determined according to their size and granularity. After doublet exclusion, CD3^+^ T cells were selected with subsequent gating to identify CD4^+^ and CD8^+^ subsets. IFN-γ^+^, IL-17A/IL-17-F^+^, IL-4/IL-5/IL-13^+^ and IL-22^+^ event frequencies were established, and the number of cytokine-producing cells was reported as a percentage of the parent (CD4^+^/CD8^-^ or CD4^−^/CD8^+^) population following subtraction of the background signal observed in the media-stimulated (negative control) condition. Boolean gating was applied to assess the following CD4^+^ T cell populations: IL-17A/IL-17F^+^, IL-22^-^; IL-17A/IL-17F^-^, IL-22^+^ and; IL-17A/IL-17F^+^, IL-22^+^.

### Serological and mucosal antibody assays

The concentration of IgG and IgA in serum, and SIgA in nasal secretions with specificity to (i) *B. pertussis* whole-cell extracts (WCE), (ii) PRN, (iii) FHA, (iv) serotype 2 and 3 fimbriae (Fim2/3), and (v) PT were measured by electrochemiluminescence as described in ref. ^13^ and were standardised against the WHO International Standard Human Pertussis Antiserum 06/140. Human IgA kit (Meso Scale Diagnostics) was employed to measure total SIgA, which was used to normalise antigen-specific SIgA. All antibody concentration data are published elsewhere^13^ and were herein used to perform additional analyses.

### Masking

Immune assays were performed by laboratory personnel who were blinded to participant allocation and *B. pertussis* colonisation status post-challenge. Immunological data, including antibody and CD4^+^ T cell data, were derived in full prior to database lock. CD8^+^ T cell response data were generated post-hoc.

### Statistical analyses

Statistical analyses were performed using GraphPad Prism software (version 10.4.2). The Shapiro–Wilk test was used to assess normality. Parametrically distributed data were presented as mean (±SD), and non-parametrically distributed data were presented as median (range or IQR). Non-parametric paired data were analysed using the Friedman test with Dunn’s multiple comparisons post-hoc test, or using the two-tailed Wilcoxon matched-pairs signed-rank test with adjustment for multiplicity of testing using manual Bonferroni correction. Correlation analyses for parametric and non-parametric data were assessed using Pearson’s R and Spearman’s rho (*r*_*s*_), respectively, with adjustment for multiplicity of testing using manual Bonferroni correction where indicated. *B. pertussis* CFU ml^−1^ data were log_10_ transformed where necessary to enable visualisation of correlation data as dot plots. All analyses were exploratory as per protocol.

## Results

### Study population

**Fig. 1:**
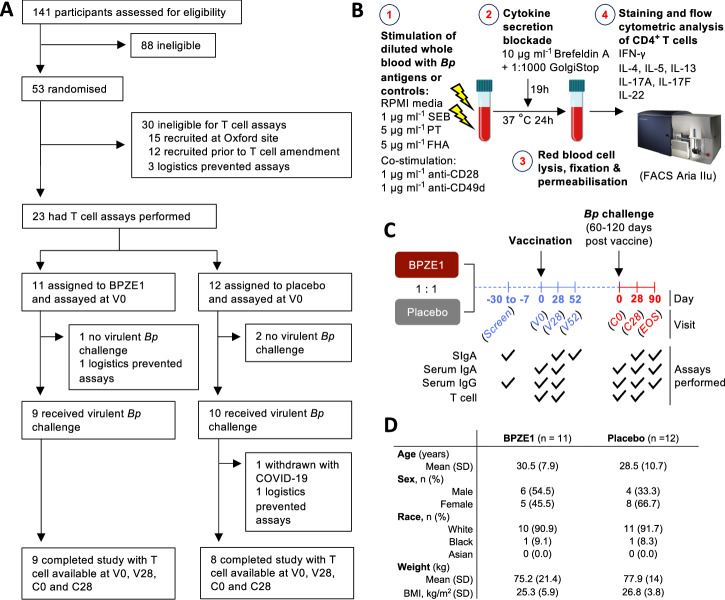
Application of whole blood T cell assay amongst vaccine-challenge trial participants. **A** Allocation of study participants where T cell assays were performed. **B** Cartoon summarising the whole blood T cell assay performed. *Staphylococcus* enterotoxin B (SEB), heat-inactivated pertussis toxin (PT), filamentous haemagglutinin (FHA). **C** Participant sampling timeline. Day of study visit relative to vaccination (blue text) or challenge with virulent *B. pertussis* (*Bp*) (red text) with corresponding visit name (‘V’ = vaccine, ‘C’ = challenge). End of study (EOS), secretory IgA (SIgA). **D** Baseline characteristics amongst participants where T cell assays were performed.

The whole blood T cell assay was performed per-protocol for exploratory analysis in a planned subset of study subjects (23 out of 53 participants) randomised to receive either BPZE1 or placebo (Fig. 1A, B), with T cell data available prior to (V0) and 28 days following (V28) vaccination for 22 of 23 participants (11 in BPZE1 group, 11 in placebo group) (see Fig. 1C for study schedule, including sampling timepoints). All study subjects in this trial had been primed in infancy with whole-cell pertussis vaccines (wP). Of the 53 participants who underwent randomisation, 46 progressed to virulent *B. pertussis* challenge at 2–4 months post-vaccination, with 17 having the T cell assays performed prior to (C0) and 28 days (C28) following challenge (9 in BPZE1 group, 8 in placebo group). Baseline characteristics of participants where the T cell assay was performed were similar between groups (Fig. 1D).

### Effector phenotype of *B. pertussis*-specific T cell responses pre-vaccination

**Fig. 2:**
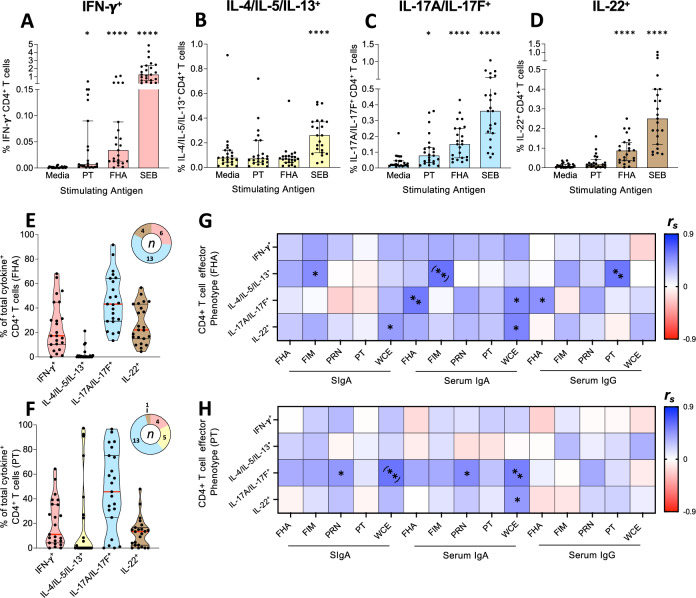
Frequency and effector phenotype of FHA- and PT-specific CD4^+^ T cell responses at baseline. The frequency of IFN-γ^+^ (pink bars) (**A**), IL-4/IL-5/IL-13^+^ (yellow bars) (**B**), IL-17A/IL-17F^+^ (blue bars) (**C**) and IL-22^+^ (brown bars) (**D**) CD4^+^ T cells with specificity to pertussis toxin (PT), filamentous haemagglutinin (FHA) and *Staphylococcus* enterotoxin B (SEB, positive control) was established using the whole blood stimulation assay at baseline (*n* = 23). Cytokine^+^ events expressed as % of the total CD4^+^ T cell population were compared with media stimulation alone using Friedman’s test with Dunn’s multiple comparisons test (*P < 0.05, ****P < 0.0001). Columns show medians, and error bars represent IQR. Violin plots showing percentage of total cytokine^+^ CD4^+^ T cells that were IFN-γ^+^, IL-17A/IL-17F^+^, IL-22^+^ and IL-4/IL-5/IL-13^+^ following stimulation with FHA (**E**) or PT (**F**), with media signal subtracted. The red line shows the median, and the error bars represent the IQR/range. Doughnut plots represent the predominant CD4^+^ effector phenotype observed across participants, expressed as participant *n*. Heat maps outlining correlation analyses comparing the frequency of FHA-specific (**G**) and PT-specific (**H**) CD4^+^ T cells vs concentrations of secretory IgA (SIgA) and serum IgA/IgG with specificity to FHA, fimbriae 2/3 (FIM), pertactin (PRN), PT and *Bordetella pertussis* whole-cell extract (WCE) at baseline. Spearman Rho (*r*_*s*_) values presented with unadjusted *P*-values, *P < 0.05, **P < 0.01. Brackets around star(s) (*) denote *P* < 0.05 following adjustment for multiplicity of testing using manual Bonferroni correction (number of tests = 15).

The frequency and effector phenotype of peripheral blood T cells with specificity to FHA and PT were assessed at baseline (V0) amongst 23 participants. At this time point, compared with media stimulation alone, stimulation of whole blood with either FHA or PT resulted in significantly higher percentages of IFN-γ^+^ and IL-17A/IL-17F^+^ CD4^+^ T cells. Stimulation with FHA also resulted in a significantly higher percentage of IL-22^+^ CD4^+^ cells than media stimulation. Neither antigen stimulated production of IL-4/IL-5/IL-13 in CD4^+^ T cells (Fig. 2A–D).

Whilst predominant polarisation signatures differed across participants, IL-17A/IL-17F^+^ responses accounted for the largest proportion of cytokine^+^ CD4^+^ T cells following FHA (Fig. 2E) or PT (Fig. 2F) stimulation at the population level. Significant positive correlations were identified between the baseline frequencies of FHA- (Fig. 2G) and PT- (Fig. 2H) specific CD4^+^ T cells and the concentrations of various types of *B. pertussis*-specific antibodies.

Stimulation with FHA, but not PT, led to a significantly higher proportion of IFNγ^+^ CD8^+^ T cells and IL-17A/IL-17F^+^ CD8^+^ T cells than media stimulation. For FHA, IFN-γ^+^ responses predominated and correlated positively in magnitude with FHA-specific IFN-γ^+^ CD4^+^ responses (Supplementary Fig. 2).

### Effect of BPZE1 on *B. pertussis*-specific circulating T cells

**Fig. 3:**
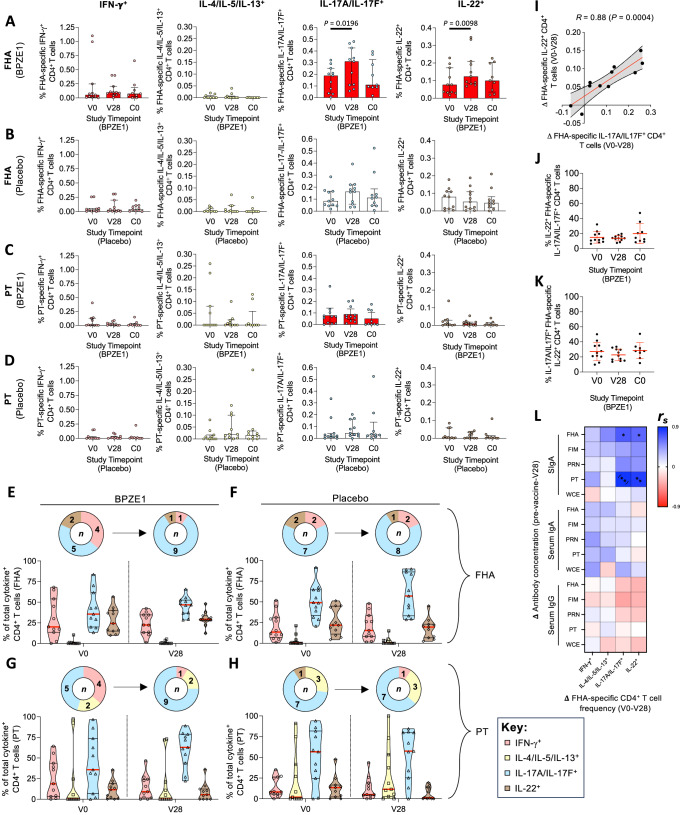
Frequency and effector phenotype of CD4^+^ T cell responses induced by BPZE1 vaccination. Filamentous haemagglutinin (FHA)-specific and pertussis toxin (PT)-specific CD4^+^ T cell frequencies that were IFN-γ^*+*^ (pink dots), IL-4/IL-5/IL-13^+^ (yellow dots), IL-17A/IL-17F^+^ (blue dots) or IL-22^+^ (brown dots) were established using the whole blood stimulation assay prior to vaccination (V0), 28 days following vaccination (V28), and prior to virulent *B. pertussis* challenge (C0) amongst participants assigned to BPZE1 (red bars) (**A**, **C**) or placebo (white bars) (**B**, **D**). Data are media subtracted. Columns show medians, error bars denote IQR. Cytokine^+^ CD4^+^ T cell frequencies compared using the Wilcoxon matched-pairs signed-rank test with manual Bonferroni correction, comparing V0 with V28, and V28 with C0 (number of tests = 2). Adjusted *P*-values shown. T cell data available for *n* = 11 (V0, V28) and *n* = 9 (C0) participants assigned to BPZE1, and *n* = 11 (V0, V28) and *n* = 10 (C0) participants assigned to placebo. Violin plots showing percentage of total cytokine^+^ CD4^+^ T cells that were IFN-γ^+^, IL-17A/IL-17F^+^, IL-22^+^ or IL-4/IL-5/IL-13^+^ following stimulation with FHA or PT, comparing V0 with V28 amongst BPZE1 and placebo groups. Doughnut plots represent the most abundant CD4^+^ effector phenotype observed across participants, expressed as participant *n* (**E**–**H**). Correlation plots with linear regression line showing association between ΔFHA-specific IL-17A/IL-17F^+^ and IL-22^+^ CD4^+^ T cells (V0-V28). Pearson’s *R* is presented with an associated *P*-value (**I**). Percentage (%) of FHA-specific IL-17A/IL-17F^+^ CD4^+^ T cells that were IL-22^+^ (**J**) and % or FHA-specific IL-22^+^ CD4^+^ T cells that were IL-17A/IL-17F^+^ (**K**) amongst BPZE1-vaccinated participants at visits V0, V28 and C0. Error bars indicate the mean with SD. Heat map outlining correlation analyses comparing the absolute change (Δ) in *B. pertussis*-specific immunoglobulin titres vs Δ FHA-specific CD4^+^ T cell frequencies (V0-V28) amongst BPZE1-vaccinated participants. Serum IgG and IgA titres were assessed between V0-V28. SIgA titres assessed between screening visit and V28. Unadjusted Spearman Rho (*r*_*s*_) values presented, *P* < 0.05*, *P* < 0.01**. Brackets around stars (*) denote *P* < 0.05 following adjustment for multiplicity of testing using manual Bonferroni correction (number of tests = 15) (**L**).

Amongst participants assigned to receive BPZE1, a significant increase in FHA-specific IL-17A/IL-17F^+^ and IL-22^+^ CD4^+^ cells was observed between V0 and V28 (Fig. 3A), which was not seen in the placebo group (Fig. 3B). That these increases occurred in response to the vaccination and were not artefacts of assay variation is substantiated by the fact that signals generated at either time point in response to media or SEB stimulation were stable over time (Supplementary Figs. 3 and4). Further analysis using Boolean gating revealed significant increases for FHA-specific IL-17^+^/IL-22^-^, IL-17^-^/IL-22^+^ and IL-17^+^/IL-22^+^ CD4^+^ T cell populations amongst BPZE1 recipients, suggesting induction of distinct Th17 and Th22 responses (Supplementary Fig. 5). No significant increase was observed in the frequency of PT-specific CD4^+^ T cells of any polarity between V0-V28 in either the BPZE1-vaccinated (Fig. 3C and Supplementary Fig. 5C) or placebo (Fig. 3D and Supplementary Fig. 5D) groups.

Consistent with these findings, there was an increase, albeit not significant, in the proportion of total cytokine-producing FHA-specific CD4^+^ T cells that were IL-17A/IL-17F^+^ and IL-22^+^ between V0-V28 amongst BPZE1 recipients (Fig. 3E), which resulted in an increase in the number of participants with FHA-specific IL-17A/IL-17F^+^ CD4^+^ T cells as the predominant effector phenotype (Fig. 3E, doughnut plots). In contrast, there was only minimal realignment of the FHA-specific T cell polarity in the placebo group (Fig. 3F). Interestingly, a non-significant increase in the proportion of IL-17A/IL-17F^+^ CD4^+^ T cells in response to PT stimulation was also observed amongst BPZE1 recipients (Fig. 3G), which was associated with an increased number of participants with PT-specific IL-17A/IL-17F^+^ CD4^+^ T cells as the predominant effector phenotype (Fig. 3G, doughnut plots), which was not seen in the placebo group (Fig. 3H). These data suggest that, although vaccination did not alter the absolute frequency of circulating PT-specific CD4^+^ T cells, it may have altered the relative composition of PT-specific CD4^+^ effector phenotypes.

The magnitude of the change (Δ) in the frequency of FHA-specific IL-17A/IL-17F^+^ CD4^+^ T cells between V0 and V28 correlated strongly with Δ in the frequency of FHA-specific IL-22^+^ CD4^+^ T cells between V0 and V28 (Fig. 3I), suggesting linkage of these responses. Importantly, only 15.5% (mean, range 6.14–47.37%) of IL-17A/IL-17F^+^ CD4^+^ T cells were double positive for IL-22 between V0 and C0 (Fig. 3J), while 26.0% (9.76–50.72%) of IL-22^+^ CD4^+^ events were double positive for IL-17A/IL-17F over the same period (Fig. 3K). There was no change in the frequency of circulating CD8^+^ T cells with specificity to either FHA or PT in either the BPZE1 or placebo groups (Supplementary Fig. 6).

To assess whether the Δ in the frequency of FHA-specific CD4^+^ T cells induced by BPZE1 were linked with the Δ in *B. pertussis*-specific humoral responses, we compared the absolute change in these responses over the same period (raw humoral response data previously reported^13^, see Supplementary Data File). Interestingly, significant correlations were observed between Δ FHA- and Δ PT-specific SIgA responses and Δ FHA-specific IL-17A/IL-17F^+^ and IL-22^+^ CD4^+^ T cell frequencies (Fig. 3L and Supplementary Fig. 7).

### Effect of virulent *B. pertussis* challenge on circulating T cells

**Fig. 4:**
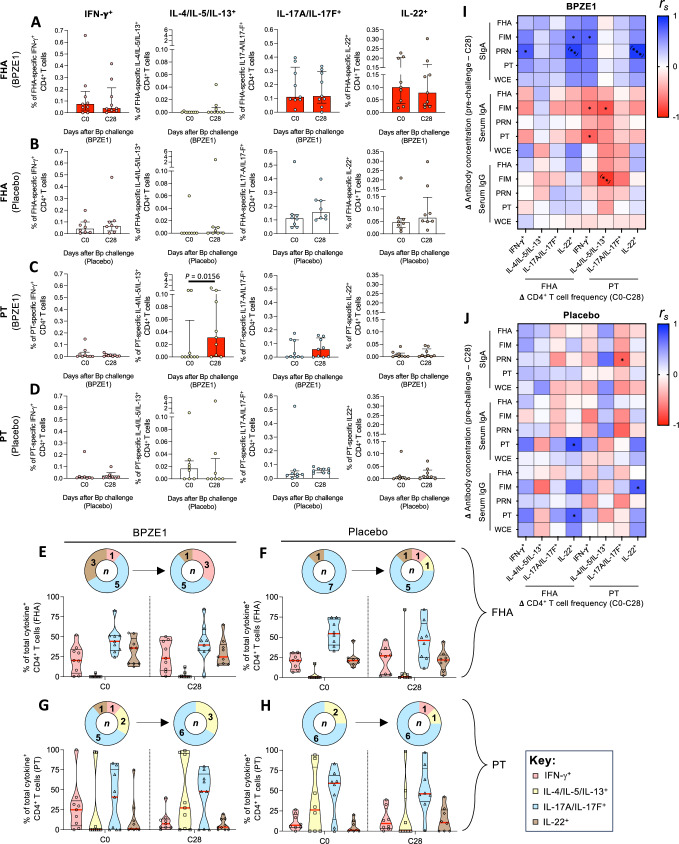
Frequency and effector phenotype of CD4^+^ T cell responses induced following virulent *B. pertussis* challenge. Filamentous haemagglutinin (FHA)-specific and pertussis toxin (PT)-specific CD4^+^ T cell frequencies that were IFN-γ^*+*^ (pink dots), IL-4/IL-5/IL-13^+^ (yellow dots), IL-17A/IL-17F^+^ (blue dots) or IL-22^+^ (brown dots) were established using the whole blood stimulation assay prior to (C0) and 28 days following (C28) intranasal challenge with virulent *B. pertussis* amongst participants assigned to BPZE1 (red bars) (**A**, **C**) or placebo (**B**, **D**) (white bars). Columns show medians, error bars denote IQR. Cytokine^+^ CD4^+^ T cell frequencies compared using the Wilcoxon matched-pairs signed-rank test. T cell data available for *n* = 9 participants assigned to BPZE1, and *n* = 8 participants assigned to placebo. Violin plots showing percentage of total cytokine^+^ CD4^+^ T cells that were IFN-γ^+^, IL-17A/IL-17F^+^, IL-22^+^ or IL-4/IL-5/IL-13^+^ following stimulation with FHA or PT, comparing C0 with C28, amongst participants in BPZE1 and placebo groups. Doughnut plots represent the most abundant CD4^+^ effector phenotype observed across participants, expressed as participant *n* (**E**–**H**). Heat maps outlining correlation analyses comparing the absolute change (Δ) in *B. pertussis*-specific immunoglobulin titres vs FHA-specific and PT-specific CD4^+^ T cell frequencies between C0 and C28 amongst participants in BPZE1 and placebo groups. Serum IgG and IgA titres assessed between C0 and C28. SIgA titres assessed between V52 (52 days post-vaccination) and C28. Spearman Rho (*r*_*s*_) values presented with unadjusted *P*-values (*P* < 0.05*. *P*, 0.01**, *P* < 0.001***). Brackets around stars (*) denote *P* < 0.05 following adjustment for multiplicity of testing using manual Bonferroni correction (number of tests = 15) (**I**, **J**).

There were no significant changes in the frequency of FHA-specific CD4^+^ T cells of any polarity in either the BPZE1-vaccinated (Fig. 4A) or placebo (Fig. 4B) groups between C0 and C28. However, there was a significant increase in the frequency of PT-specific IL-4/IL-5/IL-3^+^ CD4^+^ T cells in BPZE1 vaccinees (Fig. 4C), which was absent in the placebo group (Fig. 4D). Consistent with these findings, there was only minimal realignment of FHA-specific CD4^+^ T cells of any polarity in either group (Fig. 4E, F) but a non-significant increase in the proportion of PT-specific IL-4/IL-5/IL-13^+^ CD4^+^ T cells following virulent *B. pertussis* challenge in BPZE1-vaccinated participants (Fig. 4G), which was not seen in the placebo group (Fig. 4H).

There was no change in the frequency of circulating CD8^+^ T cells of any effector phenotype with specificity to either FHA or PT in either the BPZE1 or placebo groups following virulent *B. pertussis* challenge (Supplementary Fig. 8).

Consistent with the linkage of *B. pertussis*-specific CD4^+^ T cell and SIgA responses post-vaccination (V0-V28) (Fig. 3L), we observed that the Δ in the frequency of (i) FHA- and (ii) PT-specific CD4^+^ T cell responses following virulent *B. pertussis* challenge (C0–C28) positively correlated with Δ *B. pertussis*-specific SIgA responses over linked time periods (Fig. 4I and Supplementary Fig. 9) amongst BPZE1 recipients only. That this effect was restricted to the BPZE1 group suggests this immune dynamic, generated in response to virulent *B. pertussis*, was likely a consequence of prior BPZE1 vaccination (Fig. 4I, J).

### BPZE1-induced nasal SIgA responses as predictors of *B. pertussis* colonisation density following virulent challenge

A critical finding of our previous study was that BPZE1-vaccinated participants who developed breakthrough *B. pertussis* infection following virulent challenge had significantly reduced *B. pertussis* colonisation density and cleared *B. pertussis* infection more rapidly compared to their placebo-vaccinated counterparts^13^. To establish whether BPZE1-induced humoral or T cell responses might account for the observed effect, correlation analyses were performed comparing all immune components that increased significantly between baseline and V28 following vaccination (antibody response data are from ref. ^13^ (Supplementary Data File)) with *B. pertussis* colonisation density following virulent *B. pertussis* challenge, expressed as area under the curve (AUC, day 9–14 post-challenge, reported previously^13^). Then, to establish whether post-challenge responses were linked to *B. pertussis* colonisation density, similar analyses were performed comparing the change in *B. pertussis*-specific immune components pre- vs. post-*B. pertussis* challenge with *B. pertussis* AUC. The stated approach aimed to determine whether BPZE1-induced immune responses were associated with subsequent *B. pertussis* colonisation density post-challenge, while separating these effects from pre-existing (non-BPZE1-induced) immunity. This approach was supported by data from prior human studies demonstrating that various *B. pertussis*-specific immune responses, including serum FHA-, PRN- and PT-specific serum IgG titres, *B. pertussis*-specific SIgA titres in nasal lining fluid, and FHA- and PT-specific IL-22^+^ CD4^+^ T cell frequencies in peripheral blood, are associated with protection against colonisation following nasal challenge^16,17^. Consistent with findings from these previous studies, and as further justification to the approach taken to focus on the impact of vaccine-induced effects, in the current study we observed that certain *B. pertussis*-specific immune responses measured after vaccination but prior to virulent challenge, including FHA-specific IgG titres, predicted subsequent *B. pertussis* colonisation density amongst both BPZE1 and placebo recipients (Supplementary Figs. 10 and11). This observation was made despite participant enrolment being dependent on participants having baseline anti-PT and anti-PRN serum IgG titres ≤20 IU ml^−1^ and ≤30 IU ml^−1^, respectively.

**Fig. 5:**
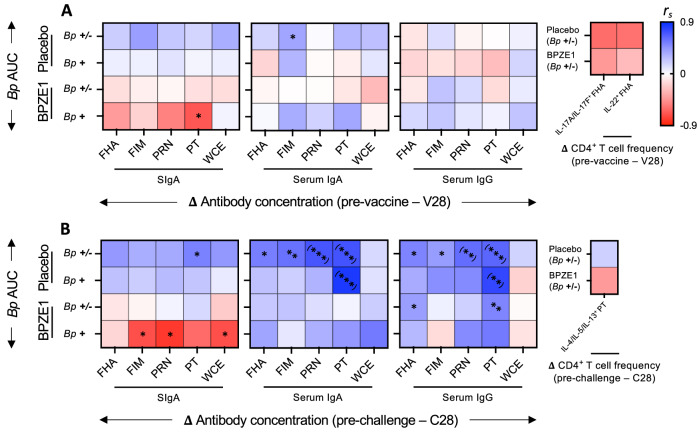
BPZE1-induced humoral and CD4^+^ T cell responses as predictors of *B. pertussis* colonisation density following virulent challenge. Heat maps outlining correlation analyses comparing *B. pertussis* (*Bp*) colonisation density (AUC, D9-14) following virulent *Bp* challenge vs. the absolute change (Δ) in *B. pertussis*-specific SIgA, serum IgA and IgG titres and CD4^+^ T cell responses following both vaccination (**A**, pre-vaccine – V28) or virulent *B. pertussis* challenge (**B**, pre-challenge – C28). Correlation analyses were performed for *B. pertussis*-challenged participants (*n* = 21 in the placebo group, *n* = 24 in the BPZE1 group), independent of subsequent colonisation status (*Bp*±) and for *Bp* colonised participants only (*Bp* +, *n* = 14 in the placebo group, *n* = 10 in the BPZE1 group), where immune assays were performed and where *Bp* AUC data were available. For the placebo group, *n* = 21 for serum IgA/IgG (V0-V28, C0–C28), *n* = 21 for SIgA (screening visit-V28), *n* = 19 for SIgA (V52–C28), *n* = 9 for CD4^+^ T cell responses (V0-V28), and *n* = 8 for T cell responses (C0–C28). For the BPZE1 group, *n* = 24 for serum IgA/IgG (V0-V28), *n* = 24 for SIgA (screening visit-V28), *n* = 23 for serum IgA/IgG (C0–C28), *n* = 22 for SIgA (V52–C28), *n* = 10 for CD4^+^ T cell responses (V0-V28), and *n* = 9 for CD4^+^ T cell responses (C0–C28). Spearman Rho (*r*_*s*_) values presented with unadjusted *P*-values. *P* < 0.05*. *P*,0.01**, *P* < 0.001***. Brackets around stars (*) denote *P* < 0.05 following adjustment for multiplicity of testing using manual Bonferroni correction (number of tests = 15).

There were no significant associations between Δ pre-vaccine (V0)-V28 *B. pertussis*-specific serum IgA/IgG titres or SIgA titres and *B. pertussis* colonisation density in the BPZE1 group overall. However, when the analysis was restricted to the *B. pertussis*-colonised group, there was a significant negative correlation between Δ pre-vaccine (screening visit)-V28 PT-specific SIgA titres and *B. pertussis* colonisation density (Fig. 5A and Supplementary Fig. 12). Following virulent *B. pertussis* challenge in the BPZE1 group, Δ pre-challenge (V52)–C28 *B. pertussis*-specific SIgA titres (FIM, PRN, WCE) negatively correlated with *B. pertussis* colonisation density, when the *B. pertussis*-colonised group was analysed. Again, no significant negative correlations were seen on comparing Δ pre-challenge (C0)–C28 *B. pertussis*-specific serum IgA/IgG titres in the BPZE1 group (Fig. 5B and Supplementary Fig. 12). The same negative correlations were not observed for participants in the placebo group, suggesting that this immune dynamic was a consequence of BPZE1 vaccination. In contrast to *B. pertussis*-specific SIgA, strong positive correlations were observed between Δ pre-post-challenge *B. pertussis*-specific serum IgA/IgG titres and *B. pertussis* colonisation density, suggesting that *B. pertussis* colonisation density directly drove the magnitude of serum antibody response generated (Fig. 5B and Supplementary Fig. 12). No significant associations were observed between the magnitude of *B. pertussis*-specific CD4^+^ T cell responses induced by either BPZE1 or virulent challenge and *B. pertussis* colonisation density. However, small numbers (10 in the BPZE1 group and 9 in the placebo group) prevented meaningful subgroup analyses (Fig. 5A, B).

## Discussion

The data reported here demonstrate for the first time that vaccination with BPZE1 induces circulating antigen-specific Th17 and Th22 CD4^+^ T cell responses in humans primed in childhood with wP vaccine. BPZE1 protects against *B. pertussis* infection in murine^8,10,18^, non-human primate^19^ and human models^13^, but the immunological mechanisms underpinning protection in humans were hitherto unexplored. In mice, BPZE1-induced protection against nasal *B. pertussis* infection is dependent upon an IL-17-dependent SIgA-based mechanism^8^, and natural infection, known to prevent subsequent infection, induces CD4^+^ Th17 tissue-resident memory T (T_RM_) cells that mediate clearance via IL-17-dependent neutrophil recruitment^20^. While it is not currently possible to determine the exact immunological mechanism(s) through which BPZE1 protects against nasopharyngeal *B. pertussis* colonisation in humans using the controlled human infection model, the aforementioned preclinical findings are consistent with immunodynamic data presented here demonstrating restricted coordination between *B.* *pertussis-*specific IL-17A/IL-17F^+^ CD4^+^ T cell and SIgA responses amongst BPZE1 recipients.

Whilst there was heterogeneity in the predominant FHA- and PT-specific CD4^+^ T cell effector phenotypes observed prior to vaccination at the individual participant level, IL-17^+^ responses were predominant across the whole cohort, consistent with wP priming^21^. Interestingly, PT-specific IFN-γ^+^, IL-17A/IL-17F^+^ and IL-22^+^ CD4^+^ T cell responses were all numerically lower than those for FHA (Fig. 2A, C and D), in contrast to findings from a previous study performed on large cohorts of children and adults using a similar whole blood simulation assay^22^. However, these differences are difficult to interpret given that participants in this study were selected for participation based on low anti-PT (≤20 IU ml^−1^) and anti-PRN (≤30 IU ml^−1^) serum IgG titres.

BPZE1-induced SIgA responses appear transient, decreasing to near baseline by 52 days following vaccination^13^, yet may still contribute to long-term control if supported by other arms of the mucosal immune response. In mice, protection persists despite rapid waning of SIgA, suggesting that long-lived plasma cells and local T cell help may sustain mucosal defence^8,23^. It is likely that SIgA secretion from long-lived plasma cells would depend upon help from IL-17-secreting CD4^+^ T_RM_ as they have been implicated in IgA plasma cell switch and upregulation of pIgR, necessary for transcytosis of IgA across the luminal surface^24,25^. In keeping with this hypothesis, here we demonstrate a restricted positive correlation between BPZE1-induced *B. pertussis*-specific IL-17^+^ and IL-22^+^ CD4^+^ cell response magnitude and SIgA titres. While the same significant positive correlations were not observed between IL-17^+^ CD4^+^ T cells and SIgA responses amongst BPZE1 recipients following virulent *B. pertussis* challenge, IL-22^+^ CD4^+^ T cells and SIgA responses remained strongly positively correlated. To the best of our knowledge, the role of Th22 cells in protection against *B. pertussis* infection has not been assessed. Therefore, further investigation is warranted to determine the role of IL-22 in protection against *B. pertussis*, given the known role of IL-22 in maintaining mucosal barrier integrity, augmenting antimicrobial peptide production, and enhancing mucosal Th1/Th22 responses^26–28^. In addition, high frequencies of FHA- and PT-specific CD4^+^ IL-22^+^ cells were recently shown to predict protection against virulent *B. pertussis* infection in the controlled human infection setting^17^.

Unexpectedly, we observed that PT-specific IL-4/IL-5/IL-13^+^ (Th2) CD4^+^ T cells increased significantly following challenge with virulent *B. pertussis* (C0–C28) amongst participants vaccinated with BPZE1. This observation is curious and, as of yet, unexplained. One plausible but speculative explanation could be that repeated rounds of upper-respiratory tract exposure to *B. pertussis* (with BPZE1 and virulent form) led to boosting of pre-existing anti-PT CD4^+^ Th2 responses, noting that in contrast to FHA responses, PT-specific CD4^+^ responses were Th2-predominant for some participants at baseline (*n* = 5/23, Fig. 2F). Note however that the impact of immune selection of participants enrolled into this trial (anti-PT and anti-PRN serum IgG titres ≤20 IU ml^−1^ and ≤30 IU ml^−1^, respectively) on this observation remains undetermined. While there is sparsity of evidence relating to induction of Th2 responses following natural *B. pertussis* infections in humans with heterogeneous baseline anti-*B. pertussis* immunity, mixed Th1/Th2 responses have previously been detected in humans post-infection^29^, while outside of the pertussis context, CD4^+^ responses to orally administered tetanus toxoid, another exotoxin, induced Th2 but not Th1 responses^30^. While the implications of PT-specific Th2 response induction observed here are as of yet unknown, it is plausible that such a response would be beneficial given the role of Th2 responses in supporting the generation of neutralising antibodies against extracellular toxins, noting that anti-PT neutralising antibody titres are critical in providing protection against severe pertussis infection^31–35^.

A critical finding of our previous study was that BPZE1-vaccinated participants who developed breakthrough *B. pertussis* infection following virulent challenge had significantly reduced *B. pertussis* colonisation density and cleared *B. pertussis* infection more rapidly compared to their placebo-vaccinated counterparts^13^. While the impact of this observation on onward transmission is uncertain, it is likely that such an effect would significantly attenuate disease, given that the clinical severity of pertussis strongly correlates with bacterial burden^36,37^. Here we have revealed that, amongst BPZE1-vaccinated subjects, the density of *B. pertussis* colonisation negatively correlated with the magnitude of *B. pertussis*-specific SIgA titres induced following both vaccination and challenge. While meaningful similar subgroup analyses for T cell responses were prohibited by low numbers, these data suggest that BPZE1-induced SIgA responses may have contributed at least in part to *B. pertussis* burden control amongst participants who developed breakthrough *B. pertussis* infection despite prior BPZE1 vaccination. While caution is required when making such an inference, it is clear that the immune dynamic outlined linking SIgA titres to reduced *B. pertussis* burden was BPZE1-induced, as the same effect was not observed amongst placebo recipients. The observation that BPZE1-induced serological responses were not linked to density of *B. pertussis* colonisation validates preclinical data suggesting that such responses, induced by either vaccination or natural infection, do not provide sterilising immunity against nasal *B. pertussis* infection^6,8^.

Although BPZE1-induced SIgA titres were negatively associated with *B. pertussis* burden amongst participants who developed breakthrough infection, no significant negative correlations were observed when assessing for effects across the entire group, including *B. pertussis* colonised and uncolonised participants. This may be due to low sample size and lack of statistical power. Alternatively, immune factors not measured in this study may be responsible for BPZE1-induced sterilising immunity in humans. Conversely, amongst placebo-vaccinated participants, we observed that *B. pertussis* colonisation density positively correlated with the magnitude of *B. pertussis*-specific SIgA, serum IgA and serum IgG responses following challenge, suggesting that *B. pertussis* colonisation burden was a direct driver of seroconversion magnitude in this group. While results from controlled human infection models using *B. pertussis* or other bacterial species that reside in the upper-respiratory tract demonstrate a clear link between colonisation status and seroconversion following challenge^7,17,38–41^, to the best of our knowledge, this is the first study to directly link early post-challenge bacterial density to the magnitude of subsequent seroconversion.

The randomised, placebo-controlled design of this study was its key strength, with all immunological assays, including derivation of data, performed in a blinded manner using fully validated assays. However, there are limitations of this analysis. First, the inability to perform the T cell assay amongst all participants resulted in small numbers of subjects in the T cell analysis set, which likely reduced statistical power and prevented meaningful subgroup analyses to establish T cell correlates with *B. pertussis* colonisation density. In addition, participants with serum anti-PT IgG titres >20 IU ml^−1^ and serum anti-PRN IgG titres >30 IU ml^−1^ were not eligible for enrolment in the trial due to the operating characteristics of the controlled human infection model utilised as the efficacy readout, noting that prior anti-*B. pertussis* immunity associates with protection against colonisation following virulent challenge^7,17^. These factors limit the generalisation of the immunological findings. Second, assessment of T cell responses was limited to those specific to PT and FHA. This approach was justified on the basis that these antigens were validated in the context of this assay prior to study start, and noting the investigatory team already had significant experience utilising these vaccine antigens with this assay^15,17^. With proof of principle established that circulating CD4^+^ T cell responses can be detected in response to BPZE1 vaccination, broader *B. pertussis* antigen pools, e.g., whole organism preparations or peptide pools, could now be utilised to expanded analyses in future studies^42,43^. Third, while we can conclude that the negative association between SIgA titres and *B. pertussis* burden observed amongst participants who developed infection despite BPZE1 vaccination was BPZE1-induced (due to the absence of a similar immunodynamic in the placebo group), it is not possible to determine whether SIgA is mechanistically responsible for reduced *B. pertussis* burden in the context of this human study. Although murine studies suggest a causal relationship^8^, we cannot exclude that another variable linked to the observed SIgA response may underpin reduced *B. pertussis* burden in this group. Fourth, the findings reported here are relevant only to wP-primed individuals, as aP-primed participants were not included in this trial (note that aP vaccination was introduced into the UK schedule in September 2004, hence individuals from this cohort were less than 18 years old at study start). However, a future planned Phase III clinical trial will definitively assess whether BPZE1 affords protection in the context of aP-primed participants. Fifth, we were unable to assess *B. pertussis*-specific CD4^+^ T_RM_ responses or mucosal neutrophil responses in this study, which have been shown to afford sterilising immunity against nasal *B. pertussis* infection in animal models^20^. While *B. pertussis*-specific T_RM_ are underexplored in primates, *B. pertussis*-specific Th17 and Th1 CD4^+^ T_RM_ have been previously detected in *B. pertussis*-infected baboons^44^, and recent methodological advances now allow their examination in humans using minimally invasive sampling^43^, opening opportunities to study upper-respiratory mucosal cellular responses and their role in vaccine-induced protection.

## Supplementary information

supplementary information

supplementary Data

## Data Availability

All data generated or analysed during this study are included in this published article and its supplementary information files. Requests for additional trial-associated data should be submitted to the corresponding author for consideration. Access to anonymised data might be granted after review.
