# Supplementary material for: BPZE1 vaccination induces IL-17+ and IL-22+ CD4+ T cells associated with nasal mucosal secretory IgA responses in humans: effects on virulent Bordetella pertussis colonisation
Source: NPJ Vaccines. 2026 Apr 18;11:121. doi: 10.1038/s41541-026-01443-7 (PMC13280450; doi:10.1038/s41541-026-01443-7)
Supplement: Supplementary file 1 — supplementary information [file 41541_2026_1443_MOESM1_ESM.pdf]

Marker	Clone	Species	Fluorochrome	Company	Reference	Validation
CD3	SK7	mouse	APC H7	BD Biosciences	641415	https://www.bdbiosciences.com/content/dam/bdb/products/global/reagents/flow-cytometry-reagents/clinical-diagnostics/single-color-antibodies-asr-ivd-ce-ivd/641xxx/6414xx/641415_base/pdf/23-5019.pdf
CD4	SK3	mouse	BB515	BD Biosciences	565996	https://www.bdbiosciences.com/content/dam/bdb/products/global/reagents/flow-cytometry-reagents/research-reagents/single-color-antibodies-ruo/565xxx/5659xx/565996_base/pdf/565996.pdf
CD8	SK1	mouse	PerCP-Cy5.5	BD Biosciences	565310	https://www.bdbiosciences.com/content/dam/bdb/products/global/reagents/flow-cytometry-reagents/research-reagents/single-color-antibodies-ruo/565xxx/5653xx/565310_base/pdf/565310.pdf
IFN-γ	B27	mouse	BV421	BD biosciences	562988	https://www.bdbiosciences.com/content/dam/bdb/products/global/reagents/flow-cytometry-reagents/research-reagents/single-color-antibodies-ruo/562xxx/5629xx/562988_base/pdf/562988.pdf
IL-4	MP-4-25D2	rat	APC	Biolegend	500812	https://d1spbj2x7qk4bg.cloudfront.net/en-gb/products/apc-anti-human-il-4-antibody-965?displayInline=true&filename=APC%20anti-human%20IL-4%20Antibody.pdf&leftRightMargin=15&pdf=true&topBottomMargin=15&v=20250227010954
IL-5	TRKF5	rat	APC	Biolegend	504305	https://d1spbj2x7qk4bg.cloudfront.net/en-gb/products/apc-anti-mouse-human-il-5-antibody-989?displayInline=true&filename=APC%20anti-mousehuman%20IL-5%20Antibody.pdf&leftRightMargin=15&pdf=true&topBottomMargin=15&v=20250316042147
IL-13	JES105A2	rat	APC	Biolegend	501908	https://d1spbj2x7qk4bg.cloudfront.net/en-gb/products/apc-anti-human-il-13-antibody-938?displayInline=true&filename=APC%20anti-human%20IL-13%20Antibody.pdf&leftRightMargin=15&pdf=true&topBottomMargin=15&v=20250316042147
IL-17A	BL168	mouse	PE	Biolegend	512306	https://d1spbj2x7qk4bg.cloudfront.net/en-gb/products/pe-anti-human-il-17a-antibody-4452?displayInline=true&filename=PE%20anti-human%20IL-17A%20Antibody.pdf&leftRightMargin=15&pdf=true&topBottomMargin=15&v=20250227010954
IL-17F	033-782	mouse	PE	BD Biosciences	561197	https://www.bdbiosciences.com/content/dam/bdb/products/global/reagents/flow-cytometry-reagents/research-reagents/single-color-antibodies-ruo/561xxx/5611xx/561197_base/pdf/561197.pdf
IL-22	22URTI	mouse	PECy7	eBiosciences	25-7229-42	https://www.thermofisher.com/order/genome-database/dataSheetPdf?producttype=antibody&productsubtype=antibody_primary&productId=25-7229-42&version=Local

Supplementary Table 1. Monoclonal antibodies utilised in whole blood T cell stimulation assay.

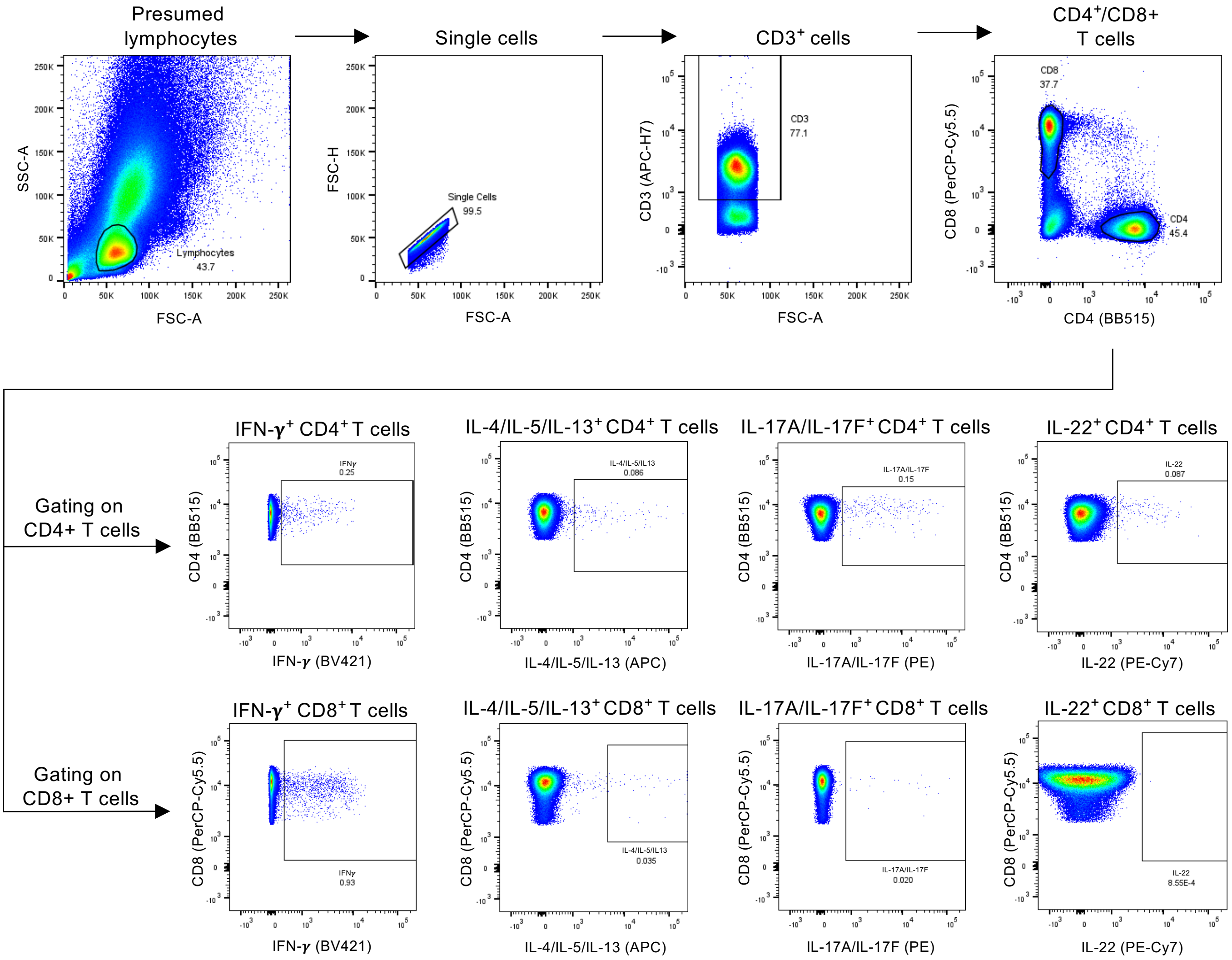

Supplementary Figure 1. Whole blood T cell stimulation assay flow cytometry gating strategy. Gating strategy utilised to determine the proportion (%, see numbers under each box indicating the positive population) of CD4+ and CD8+ T cells that were IFN-γ+, IL-4/IL-5/IL-13+, IL-17A/IL17-F+ or IL-22+. In this example, stimulation was with filamentous haemagglutinin.

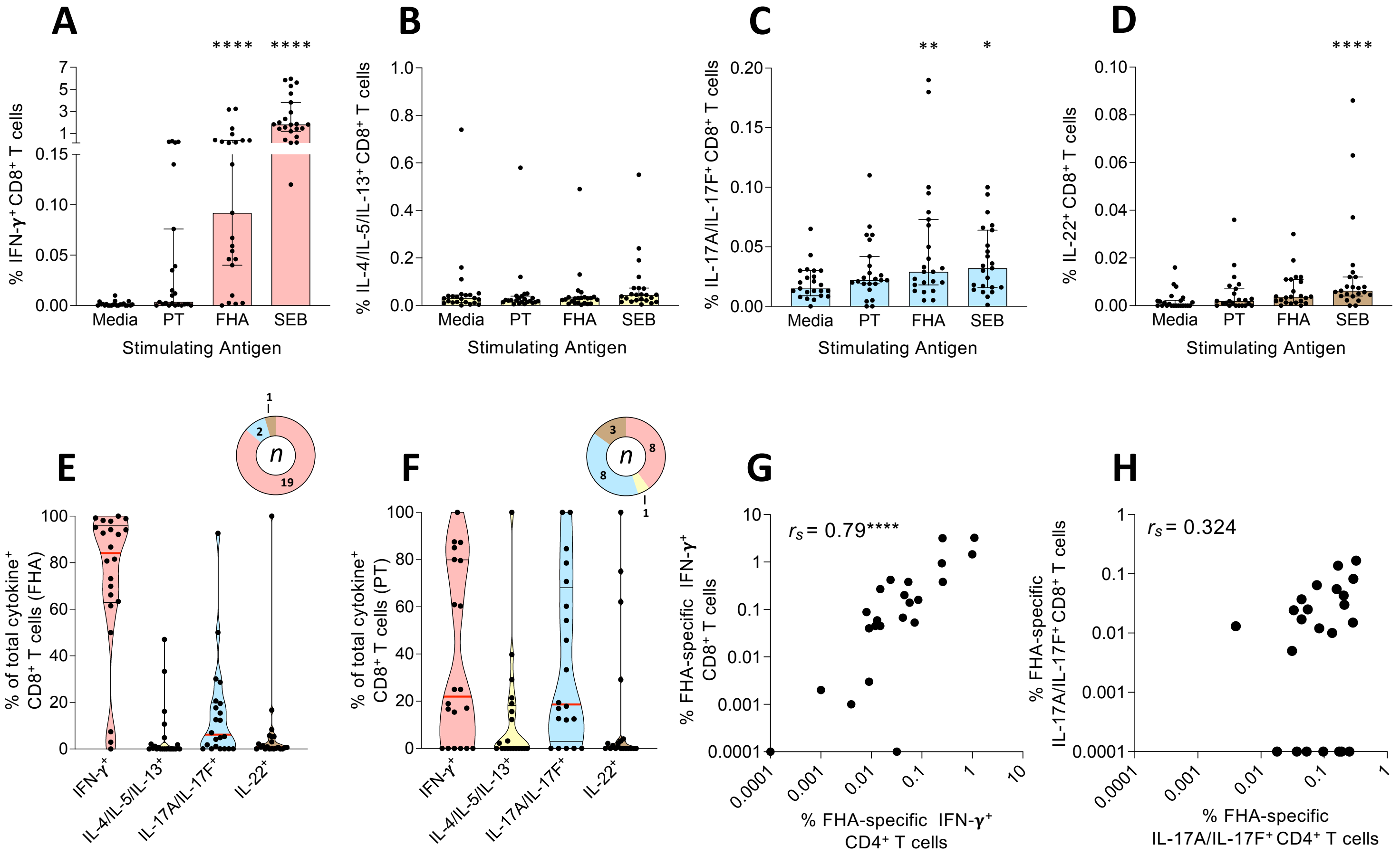

Supplementary Figure 2. Frequency and effector phenotype of FHA- and PT-specific CD8$^+$ T cell responses at baseline. The frequency of IFN-γ$^+$ (pink bars) (**A**), IL-4/IL-5/IL-13$^+$ (yellow bars) (**B**), IL-17A/IL-17F$^+$ (blue bars) (**C**), and IL-22$^+$ (brown bars) (**D**) CD8$^+$ T cells with specificity to pertussis toxin (PT), filamentous haemagglutinin (FHA) and *Staphylococcus* enterotoxin B (SEB, positive control) was established using the whole blood stimulation assay at baseline (V0, n = 23). Cytokine$^+$ events expressed as % of the total CD8$^+$ T cell population were compared with media stimulation alone using Friedman's test with Dunn's multiple comparisons test (*P <0.05, **P <0.01, ****P <0.0001). Columns show medians, error bars represent IQR. Violin plots showing percentage of total cytokine$^+$ CD8$^+$ T cells that were IFN-γ$^+$, IL-4/IL-5/IL-13$^+$, IL-17A/IL-17F$^+$ or IL-22$^+$ following stimulation with FHA (**E**) or PT (**F**), with media signal subtracted and where the total number of cytokine$^+$ CD8$^+$ T cell events was greater than zero. Red line shows median, error bars represent IQR/range. Doughnut plots represent the predominant CD8$^+$ effector phenotype observed across participants expressed as participant n. Correlation plots comparing frequencies of IFN-γ$^+$ (**G**) and IL-17A/IL-17F$^+$ (**H**) CD4$^+$ T cells vs. CD8$^+$ T cells at baseline. Spearman Rho (r_s) values presented, ****P <0.0001).

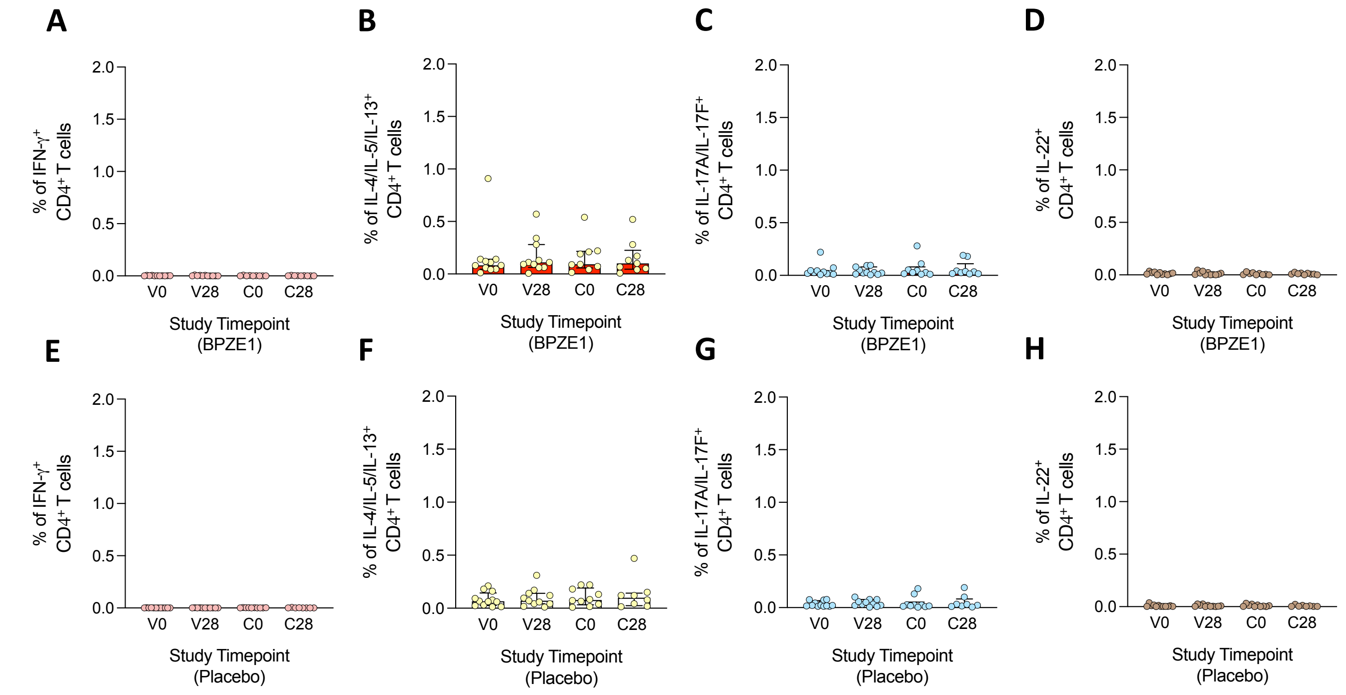

Supplementary Figure 3. Frequency and effector phenotype of CD4+ T cell responses following stimulation with media control

CD4+ T cell frequencies following mock (media only) stimulation that were IFNγ+ (pink dots), IL-4/IL-5/IL-13+ (yellow dots), IL-17A/IL-17F+ (blue dots) or IL-22+ (brown dots) were established using the whole blood stimulation assay prior to and following vaccination (V0-V28) and virulent *B. pertussis* challenge (C0-C28) amongst participants assigned to BPZE1 (red bars) or placebo (white bars). Columns show medians, error bars denote IQR. Cytokine+ CD4+ T cell frequencies compared post-vaccination using Wilcoxon matched-pairs signed rank test with manual Bonferroni correction, comparing V0 with V28, and V28 with C0 (number of tests = 2). Adjusted *P* values only shown where significant (P <0.05*). Cytokine+ CD4+ T cell frequencies compared following challenge with virulent *B. pertussis* using Wilcoxon matched-pairs signed rank test, comparing C0 with C28. *P* values shown only where significant (P <0.05*). T cell data available for n = 11 (V0, V28) and n = 9 (C0, C28) participants assigned to BPZE1, and n = 11 (V0, V28), n = 10 (C0) and n = 8 (C28) for participants assigned to placebo (**A-H**).

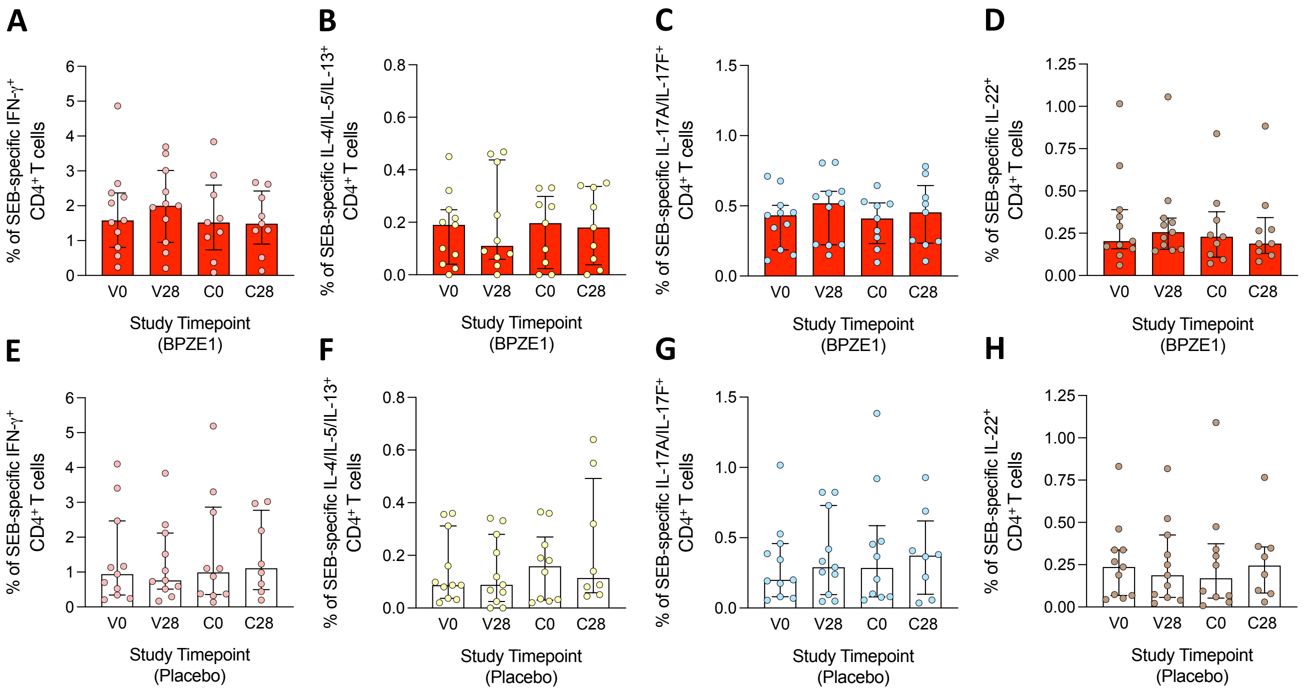

Supplementary Figure 4. Frequency and effector phenotype of CD4$^+$ T cell responses following stimulation with *Staphylococcus* enterotoxin B

Staphylococcus enterotoxin B (SEB)-specific CD4$^+$ T cell frequencies that were IFNγ^+ (pink dots), IL-4/IL-5/IL-13$^+$ (yellow dots), IL-17A/IL-17F$^+$ (blue dots) or IL-22$^+$ (brown dots) were established using the whole blood stimulation assay prior to and following vaccination (V0-V28) and virulent *B. pertussis* challenge (C0-C28) amongst participants assigned to BPZE1 (red bars) or placebo (white bars). Data are media subtracted. Columns show medians, error bars denote IQR. Cytokine$^+$ CD4$^+$ T cell frequencies compared post-vaccination using Wilcoxon matched-pairs signed rank test with manual Bonferroni correction, comparing V0 with V28, and V28 with C0 (number of tests = 2). Adjusted *P* values only shown where significant (*P* <0.05*). Cytokine$^+$ CD4$^+$ T cell frequencies compared following challenge with virulent *B. pertussis* using Wilcoxon matched-pairs signed rank test, comparing C0 with C28. *P* values shown only where significant (*P* <0.05*). T cell data available for *n* = 11 (V0, V28) and *n* = 9 (C0) participants assigned to BPZE1, and *n* = 11 (V0, V28) and *n* = 10 (C0) participants assigned to placebo (**A-H**).

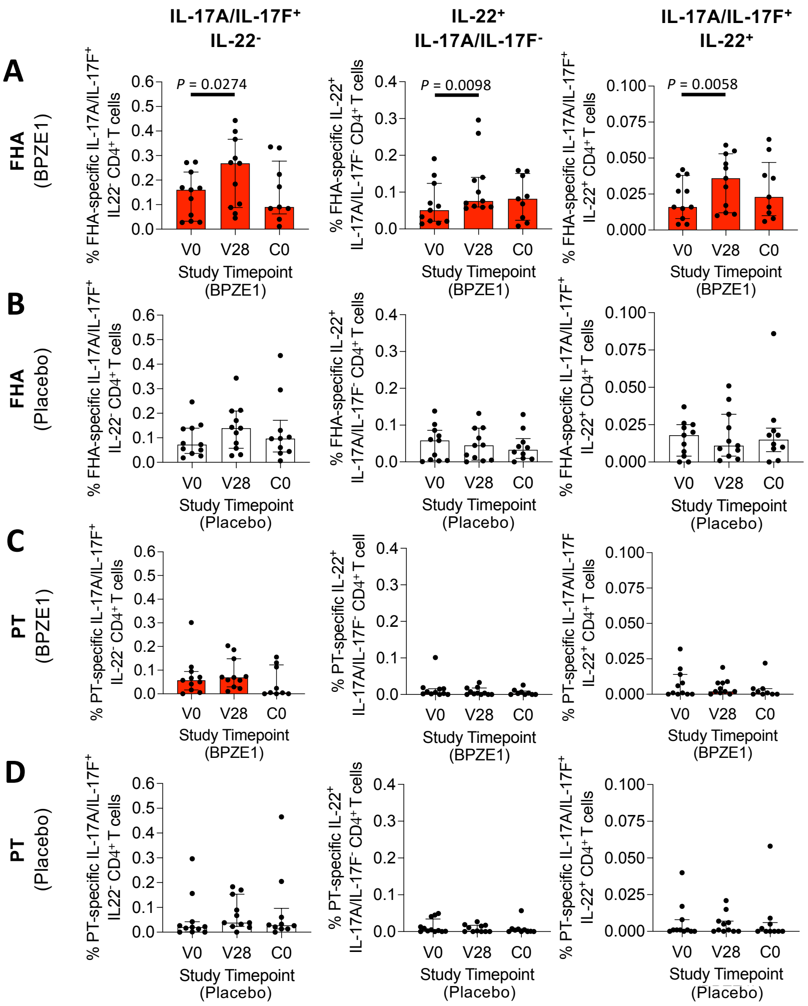

Supplementary Figure 5. Frequency of IL-17A/IL-17F⁺ IL-22⁻, IL-22⁺ IL-17A/IL-17F⁻ and IL-17A/IL-17F⁺ IL-22⁺ CD4⁺ T cell responses induced by BPZE1 vaccination. Filamentous haemagglutinin (FHA)-specific and pertussis toxin (PT)-specific CD4⁺ T cell frequencies that were IL-17A/IL17F⁺ IL-22⁻, IL-22⁺ IL-17A/IL-17F⁻ or IL-17A/IL-17F⁺ IL-22⁺ were established using the whole blood stimulation assay prior to vaccination (V0), 28 days following vaccination (V28), and prior to virulent *B. pertussis* challenge (C0) amongst participants assigned to BPZE1 (red bars) (**A,C**) or placebo (white bars) (**B,D**). Data are media subtracted. Columns show medians, error bars denote IQR. Cytokine⁺ CD4⁺ T cell frequencies compared using Wilcoxon matched pairs signed rank test with manual Bonferroni correction, comparing V0 with V28, and V28 with C0 (number of tests = 2). Adjusted *P* values shown.

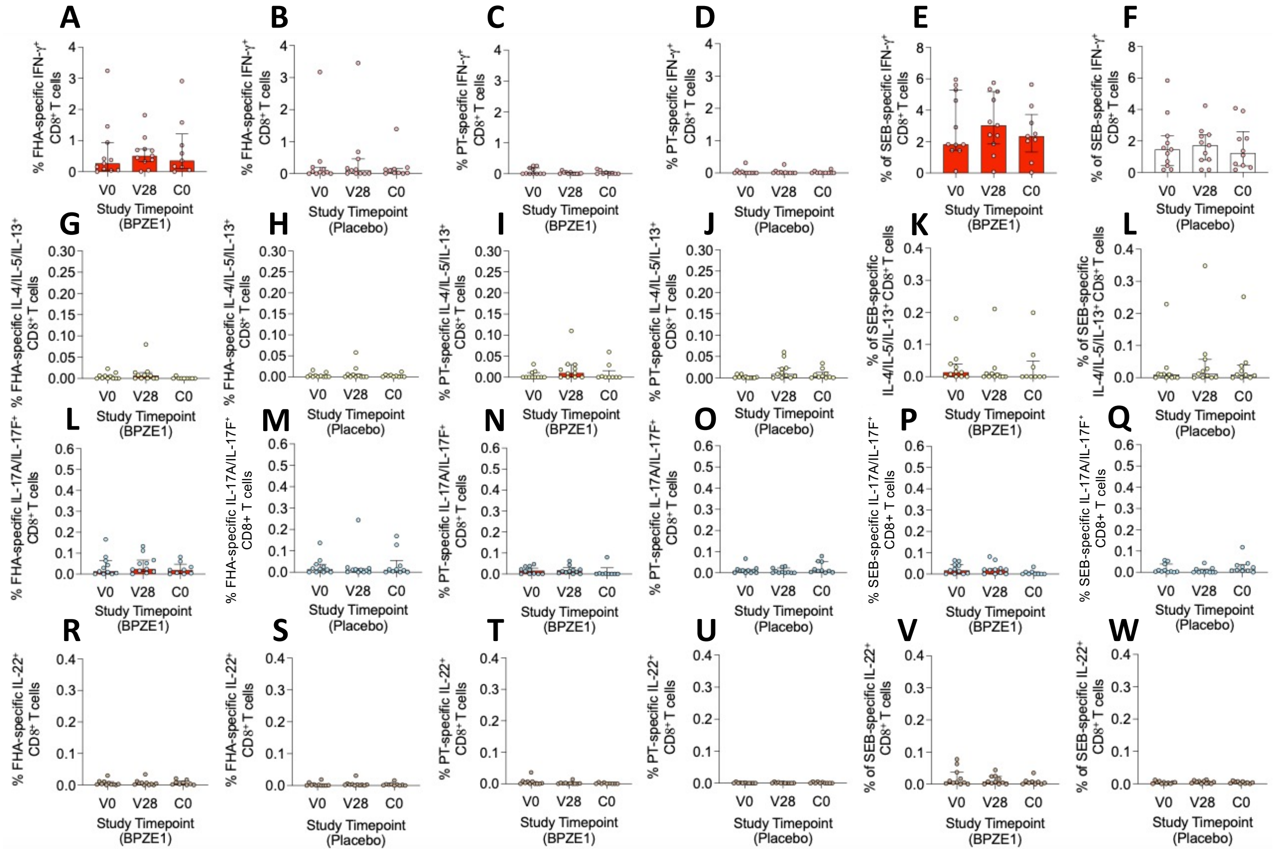

Supplementary Figure 6. Frequency and effector phenotype of CD8^{+} T cell responses induced by BPZE1 vaccination

Filamentous haemagglutinin (FHA)-specific and pertussis toxin (PT)-specific CD8^{+} T cell frequencies that were IFN-γ^{+} (pink dots, **A-D**), IL-4/IL-5/IL-13^{+} (yellow dots, **G-J**), IL-17A/IL-17F^{+} (blue dots, **L-O**) or IL-22^{+} (brown dots, **R-U**) were established using the whole blood stimulation assay prior to vaccination (V0), 28 days following vaccination (V28), and prior to virulent *B. pertussis* challenge (C0) amongst participants assigned to BPZE1 (red bars) or placebo (white bars). Corresponding responses following stimulation with *Staphylococcus* enterotoxin B (SEB, positive control) outlined in **E-F**, **K-L**, **P-Q** and **V-W**. All data are media subtracted. Columns show medians, error bars denote IQR. Cytokine^{+} CD4^{+} T cell frequencies compared using Wilcoxon matched-pairs signed rank test with manual Bonferroni correction, comparing V0 with V28, and V28 with C0 (number of tests = 2). Adjusted P values shown, where significant (P <0.05). T cell data available for n = 11 (V0, V28) and n = 9 (C0) participants assigned to BPZE1, and n = 11 (V0, V28) and n = 10 (C0) participants assigned to placebo.

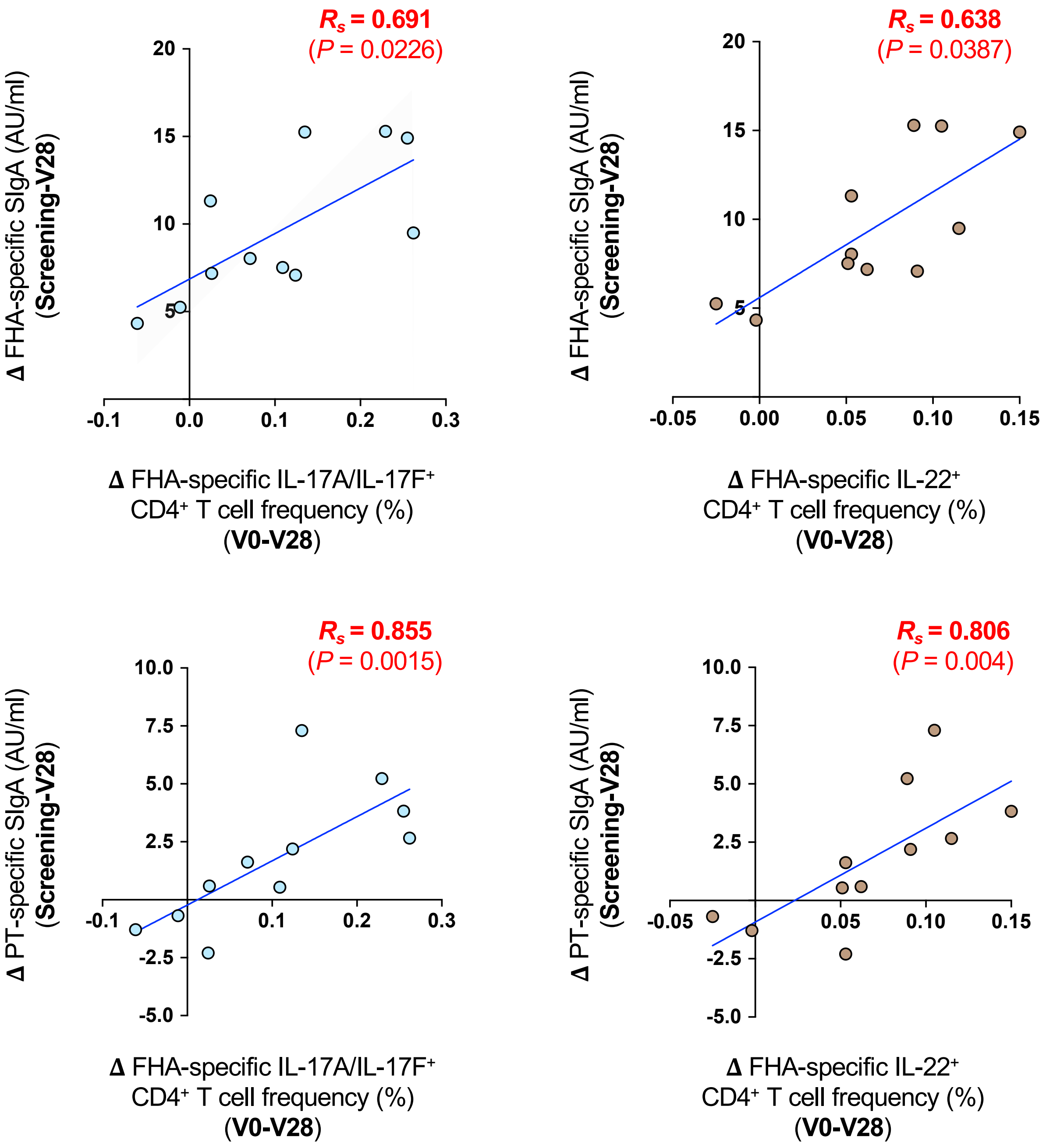

Supplementary Figure 7. Significant positive correlation analyses outlined in Figure 3L represented as dot plots. Absolute change (**Δ**) in Pertussis toxin (PT)- and Filamentous haemagglutinin (FHA)-specific secretory IgA (SIgA) titres (screening visit – V28) vs **Δ** FHA-specific $CD4^+$ T cell frequencies (V0-V28) amongst BPZE1-vaccinated participants. Unadjusted Spearman Rho (r_s) values presented with associated P value. Linear regression line included to illustrate direction of trend.

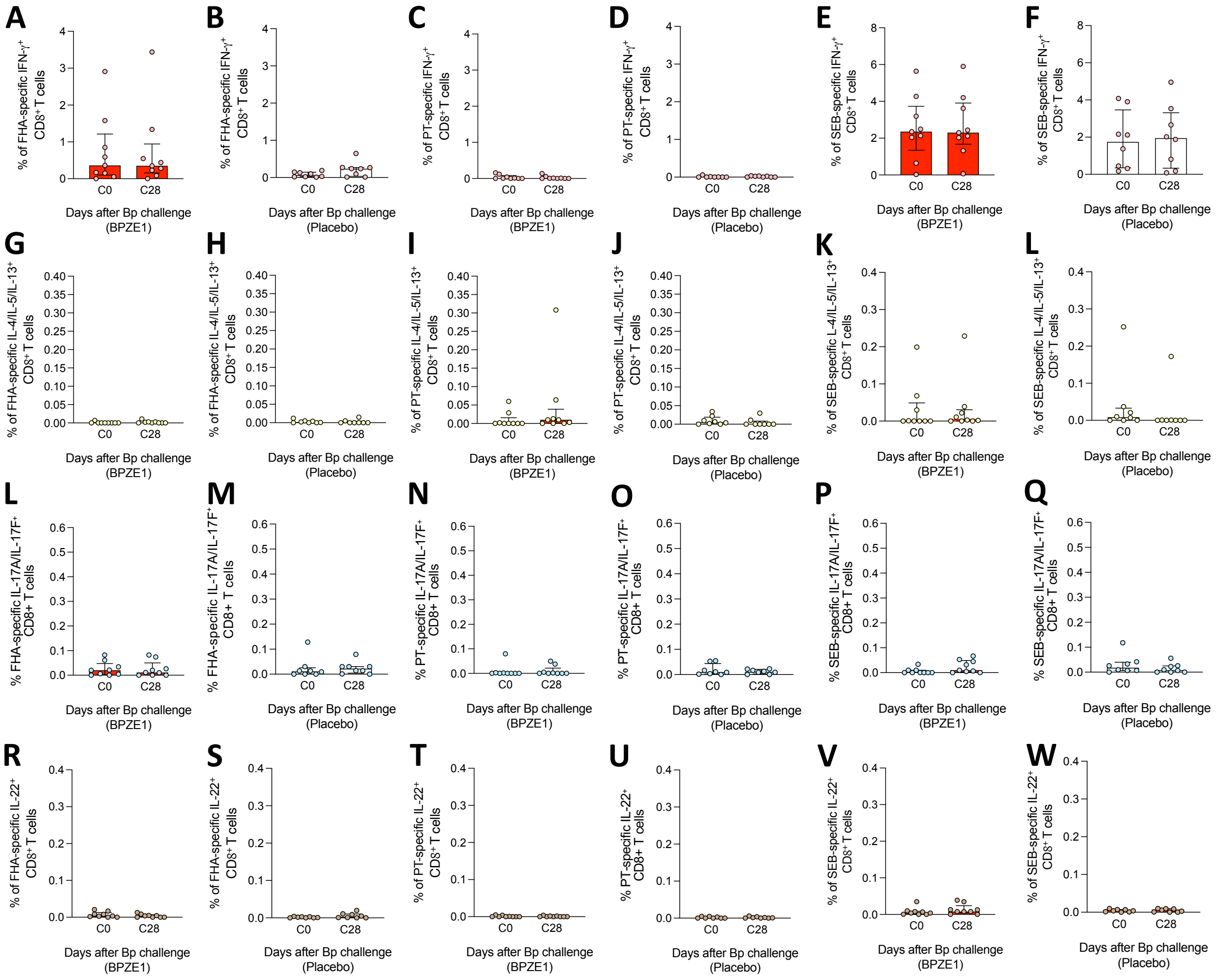

Supplementary Figure 8. Frequency and effector phenotype of CD8⁺ T cell responses induced following virulent *B. pertussis* challenge

Filamentous haemagglutinin (FHA)-specific and pertussis toxin (PT)-specific $CD8^+$ T cell frequencies that were IFN-γ^+ (pink dots, **A-D**), IL-4/IL-5/IL-13^+ (yellow dots, **G-J**), IL-17A/IL-$17F^+$ (blue dots, **L-O**) or IL-22^+ (brown dots, **R-U**) were established using the whole blood stimulation assay prior to (C0) and 28 days following (C28) challenge with virulent *B. pertussis* amongst participants assigned to BPZE1 (red bars) or placebo (white bars). Corresponding responses following stimulation with *Staphylococcus* enterotoxin B (SEB, positive control) outlined in **E-F**, **K-L**, **P-Q** and **V-W**. All data are media subtracted. Columns show medians, error bars denote IQR. Cytokine⁺ $CD4^+$ T cell frequencies compared using Wilcoxon matched-pairs signed rank test, comparing C0 with C28. Adjusted *P* values shown, where significant (P <0.05). T cell data available for n = 9 participants assigned to BPZE1, and n = 8 participants assigned to placebo.

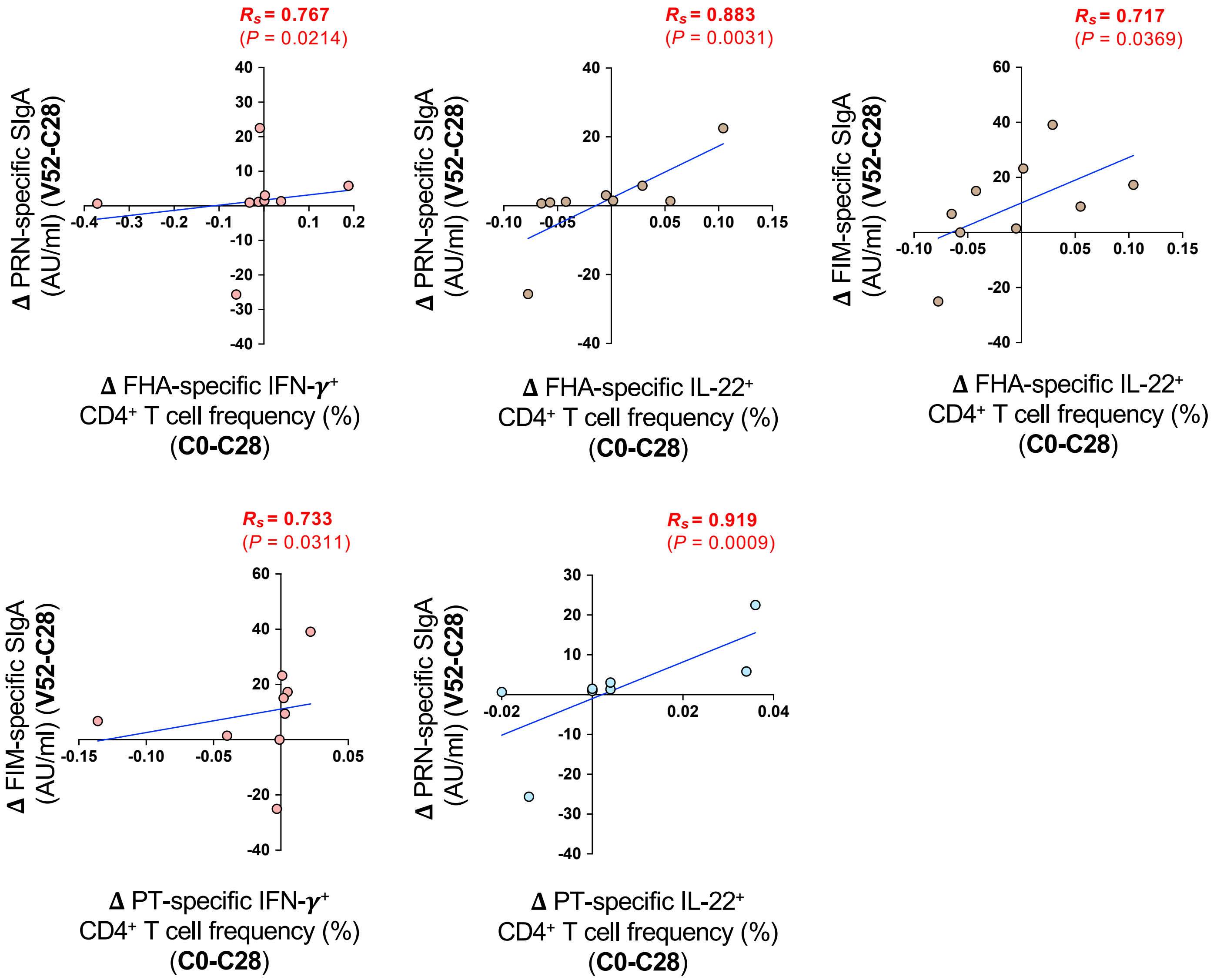

Supplementary Figure 9. Significant positive correlation analyses outlined in Figure 4I represented as dot plots. Absolute change (**Δ**) in Pertactin (PRN)- and Fimbriae (FIM)-specific secretory IgA (SIgA) titres (V52-C28) vs **Δ** Filamentous hemagglutinin (FHA) and Pertussis toxin (PT)-specific CD4+ T cell frequencies (C0-C28) amongst BPZE1-vaccinated participants. Unadjusted Spearman Rho (r_s) values presented with associated *P* value. Linear regression line included to illustrate direction of trend.

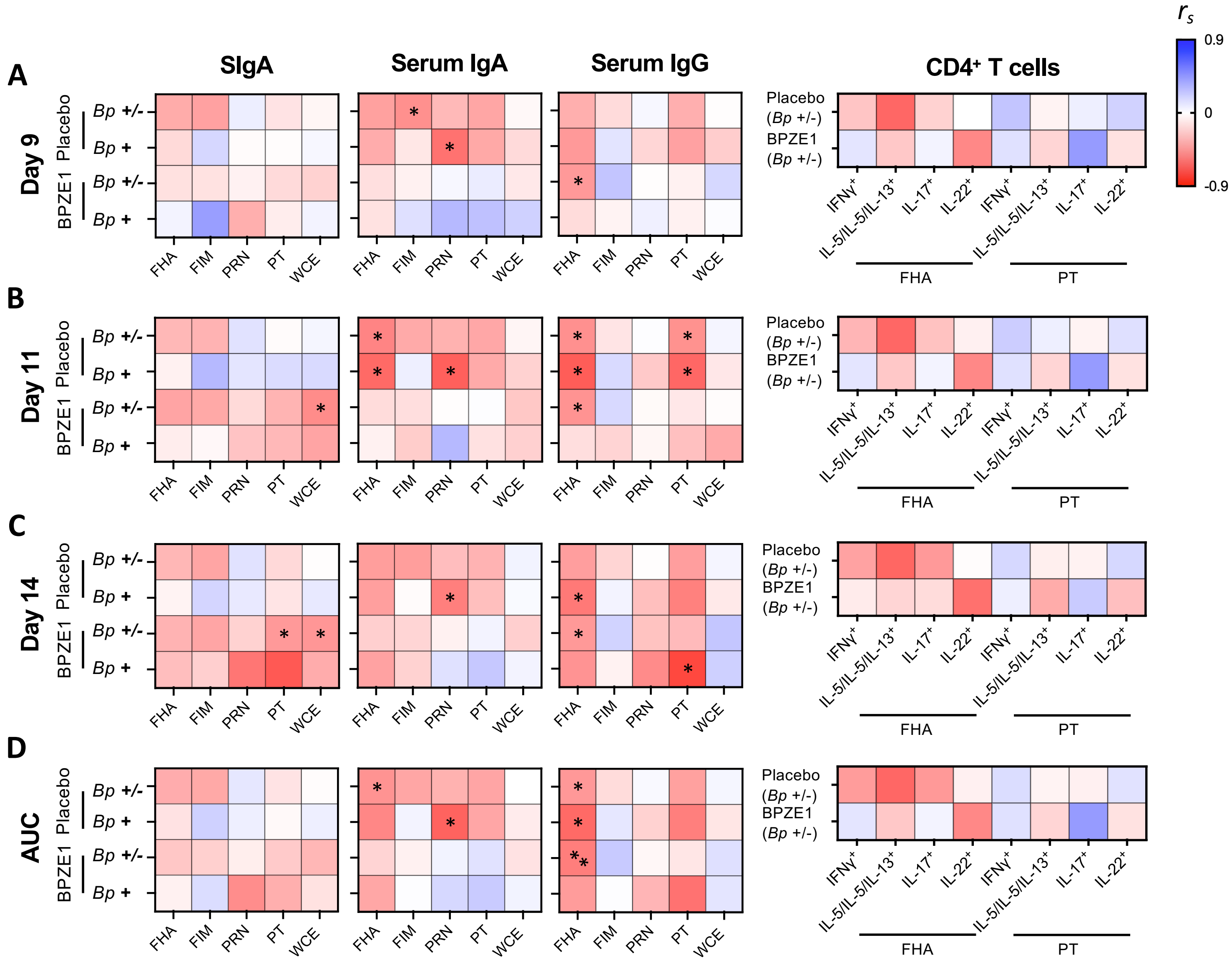

Supplementary Figure 10. Pre-challenge *B. pertussis-specific* humoral and CD4⁺ T cell responses responses as predictors of *B. pertussis* colonisation density following virulent challenge. Heat maps outlining correlation analyses comparing titres of *B. pertussis*-specific antibody concentrations and CD4⁺ T cell frequencies prior to virulent *B. pertussis* challenge vs. *B. pertussis* colonisation density at Day 9 (**A**), Day 11 (**B**), Day 14 (**C**) and AUC, D9-14 (**D**). Correlation analyses performed for *B. pertussis*-challenged participants (*n* = 21 in placebo group, *n* = 24 in BPZE1 group) independent of subsequent colonisation status (*Bp* +/-) and for *B. pertussis* colonised participants only (*Bp* +). SIgA data are from V52. Serum IgA, serum IgG and CD4⁺ T cell data are from C0. Spearman Rho (r_s) values presented with unadjusted *P* values. *P* <0.05*. *P*,0.01**. See **Supplementary Figure 11** for dot plots relating to significant correlations outlined.

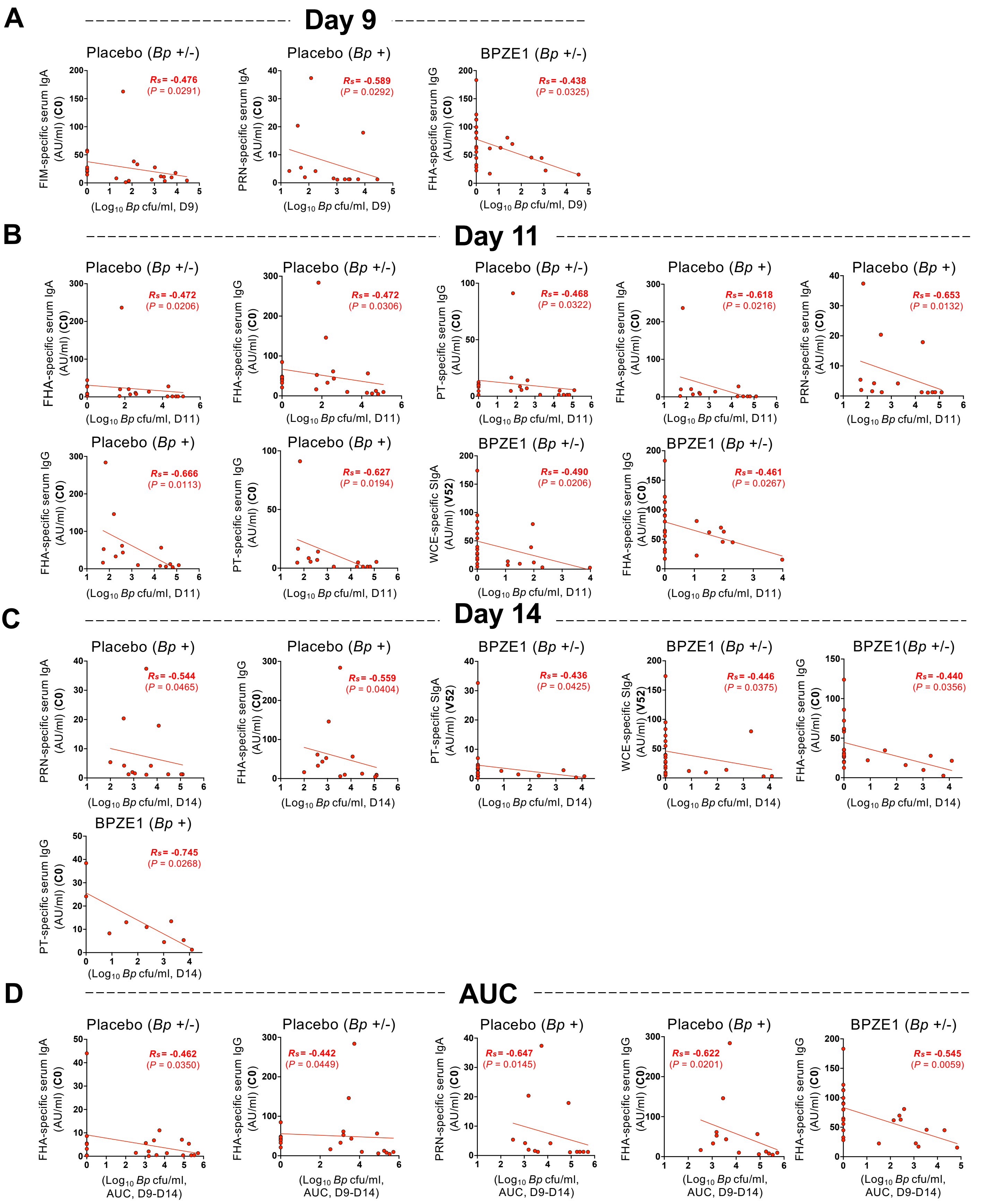

Supplementary Figure 11. Significant correlation analyses outlined in Supplementary Figure 10 heat maps represented as dot plots. *B. pertussis*-specific secretory IgA (SIgA), serum IgA and serum IgG titres vs *B. pertussis* colonisation density (Log_{10} CFU/ml) at Day 9 (D9) (**A**), Day 11 (D11) (**B**), Day 14 (D14) (**C**) and AUC (D9-14) (**D**) following challenge with virulent *B. pertussis.* Spearman Rho (r_s) values presented with associated *P* value. Linear regression line included to illustrate direction of trend. (*Bp* +) - *B. pertussis* colonised participants only. (*Bp* +/-) - All participants, independent of *B. pertussis* colonisation status. Pertussis toxin (PT), Fimbriae (FIM), Pertactin (PRN), Filamentous haemagglutinin (FHA), Whole cell extract (WCE).

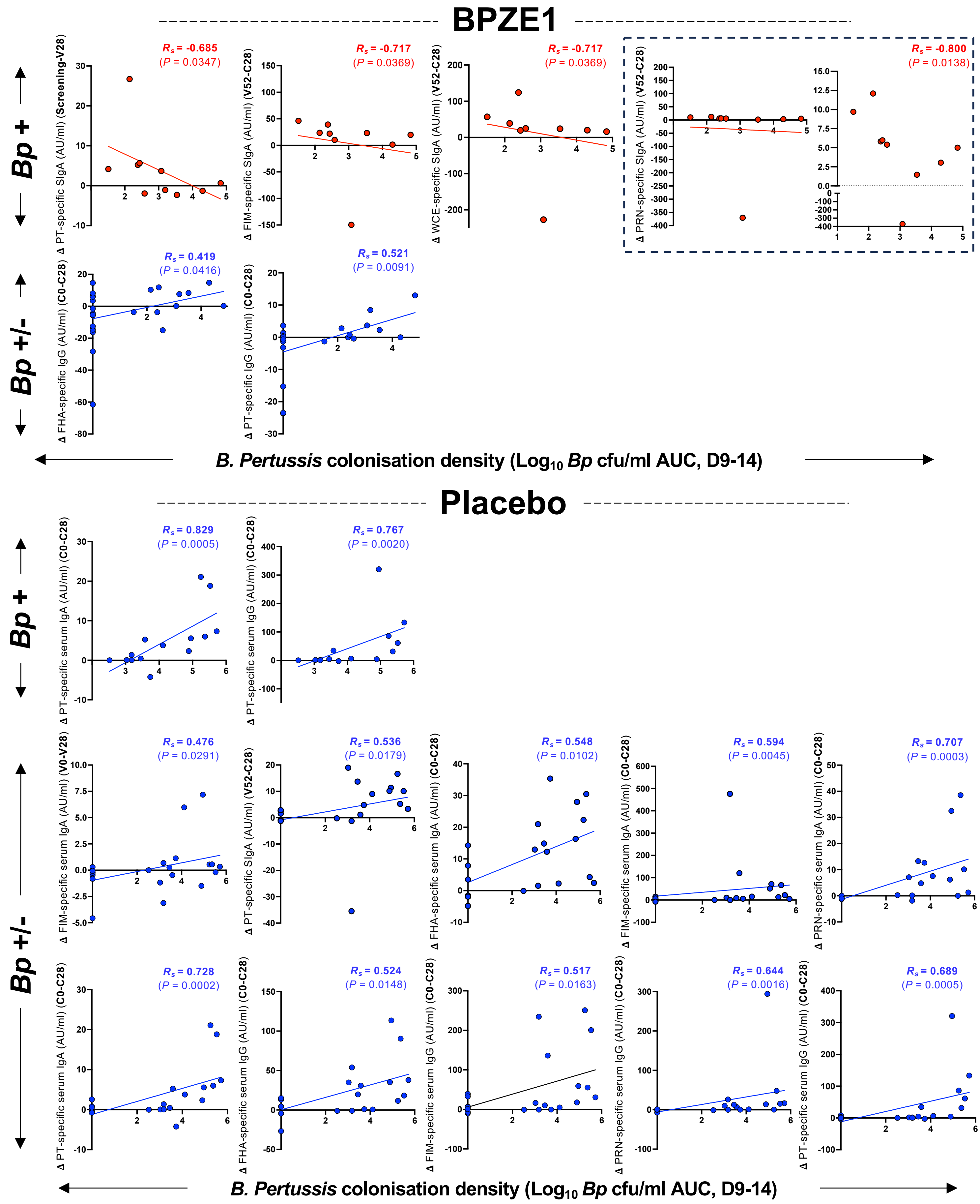

Supplementary Figure 12. Significant correlation analyses outlined in Figure 5 heat maps represented as dot plots. Absolute change (Δ) in *B. pertussis*-specific secretory IgA (SIgA), serum IgA and serum IgG titres (pre-post vaccine or pre-post challenge with virulent *B. pertussis*) vs *B. pertussis* colonisation density (Log_{10} *Bp* CFU/ml AUC, D9-14). Spearman Rho (r_s) values presented with associated *P* value. Linear regression line included to illustrate direction of trend. (*Bp* +) - *B. pertussis* colonised participants only. (*Bp* +/-) - All participants, independent of *B. pertussis* colonisation status. Pertussis toxin (PT), Fimbriae (FIM), Pertactin (PRN), Filamentous haemagglutinin (FHA), Whole cell extract (WCE).
